# 3 versus 6 months of adjuvant oxaliplatin-fluoropyrimidine combination therapy for colorectal cancer (SCOT): an international, randomised, phase 3, non-inferiority trial

**DOI:** 10.1016/S1470-2045(18)30093-7

**Published:** 2018-04

**Authors:** Timothy J Iveson, Rachel S Kerr, Mark P Saunders, Jim Cassidy, Niels Henrik Hollander, Josep Tabernero, Andrew Haydon, Bengt Glimelius, Andrea Harkin, Karen Allan, John McQueen, Claire Scudder, Kathleen Anne Boyd, Andrew Briggs, Ashita Waterston, Louise Medley, Charles Wilson, Richard Ellis, Sharadah Essapen, Amandeep S Dhadda, Mark Harrison, Stephen Falk, Sherif Raouf, Charlotte Rees, Rene K Olesen, David Propper, John Bridgewater, Ashraf Azzabi, David Farrugia, Andrew Webb, David Cunningham, Tamas Hickish, Andrew Weaver, Simon Gollins, Harpreet S Wasan, James Paul

**Affiliations:** a Southampton University Hospital NHS Foundation Trust, Southampton, UK; b Department of Oncology, University of Oxford, Oxford, UK; c The Christie Hospital, Manchester, UK; d Cancer Research UK Clinical Trials Unit, Institute of Cancer Sciences, University of Glasgow, Glasgow, UK; e Department of Oncology and Palliative Care, Zealand University Hospital, Naestved, Denmark; f Vall d'Hebron University Hospital and Institute of Oncology, Universitat Autònoma de Barcelona, CIBERONC, Barcelona, Spain; g Australasian Gastro-Intestinal Trials Group, Melbourne, VIC, Australia; h IGP, University of Uppsala, Uppsala, Sweden; i OCTO, University of Oxford, Department of Oncology, Oxford, UK; j Institute of Health and Wellbeing, University of Glasgow, Glasgow, UK; k Center for Health and Policy Outcomes, Department of Epidemiology and Biostatistics, Memorial Sloan-Kettering Cancer Centre, New York, NY, USA; l Beatson West of Scotland Cancer Centre, Glasgow UK; m Royal United Hospital, Bath, UK; n Addenbrookes Hospital, Cambridge, UK; o Royal Cornwall Hospital NHS Trust, Truro, UK; p St Luke's Cancer Centre, Royal Surrey County Hospital NHS Foundation Trust, Guildford, UK; q Castle Hill Hospital, Hull, UK; r Mount Vernon Cancer Centre, Northwood, UK; s Bristol Cancer Institute, Bristol, UK; t Barking Havering and Redbridge University Hospital NHS Trust, Barking, UK; u Department of Oncology, Aarhus University Hospital, Aarhus, Denmark; v Barts Cancer Institute, Queen Mary University of London, London, UK; w UCL Cancer Institute, University College London, London UK; x Newcastle upon Tyne Hospitals NHS Foundation Trust, Newcastle, UK; y Gloucestershire Oncology Centre, Cheltenham General Hospital, Cheltenham, UK; z Brighton and Sussex University Hospital Trust, Brighton, UK; aa Royal Marsden Hospital, London, UK; ab Poole Hospital, Bournemouth University, Bournemouth, UK; ac Department of Oncology, Oxford University Hospitals Foundation Trust, Oxford, UK; ad North Wales Cancer Treatment Centre, Rhyl, UK; ae Hammersmith Hospital, Imperial College London, London, UK

## Abstract

**Background:**

6 months of oxaliplatin-containing chemotherapy is usually given as adjuvant treatment for stage 3 colorectal cancer. We investigated whether 3 months of oxaliplatin-containing chemotherapy would be non-inferior to the usual 6 months of treatment.

**Methods:**

The SCOT study was an international, randomised, phase 3, non-inferiority trial done at 244 centres. Patients aged 18 years or older with high-risk stage II and stage III colorectal cancer underwent central randomisation with minimisation for centre, choice of regimen, sex, disease site, N stage, T stage, and the starting dose of capecitabine. Patients were assigned (1:1) to receive 3 months or 6 months of adjuvant oxaliplatin-containing chemotherapy. The chemotherapy regimens could consist of CAPOX (capecitabine and oxaliplatin) or FOLFOX (bolus and infused fluorouracil with oxaliplatin). The regimen was selected before randomisation in accordance with choices of the patient and treating physician. The primary study endpoint was disease-free survival and the non-inferiority margin was a hazard ratio of 1·13. The primary analysis was done in the intention-to-treat population and safety was assessed in patients who started study treatment. This trial is registered with ISRCTN, number ISRCTN59757862, and follow-up is continuing.

**Findings:**

6088 patients underwent randomisation between March 27, 2008, and Nov 29, 2013. The intended treatment was FOLFOX in 1981 patients and CAPOX in 4107 patients. 3044 patients were assigned to 3 month group and 3044 were assigned to 6 month group. Nine patients in the 3 month group and 14 patients in the 6 month group did not consent for their data to be used, leaving 3035 patients in the 3 month group and 3030 patients in the 6 month group for the intention-to-treat analyses. At the cutoff date for analysis, there had been 1482 disease-free survival events, with 740 in the 3 month group and 742 in the 6 month group. 3 year disease-free survival was 76·7% (95% CI 75·1–78·2) for the 3 month group and 77·1% (75·6–78·6) for the 6 month group, giving a hazard ratio of 1·006 (0·909–1·114, test for non-inferiority p=0·012), significantly below the non-inferiority margin. Peripheral neuropathy of grade 2 or worse was more common in the 6 month group (237 [58%] of 409 patients for the subset with safety data) than in the 3 month group (103 [25%] of 420) and was long-lasting and associated with worse quality of life. 1098 serious adverse events were reported (492 reports in the 3 month group and 606 reports in the 6 month group) and 32 treatment-related deaths occurred (16 in each group).

**Interpretation:**

In the whole study population, 3 months of oxaliplatin-containing adjuvant chemotherapy was non-inferior to 6 months of the same therapy for patients with high-risk stage II and stage III colorectal cancer and was associated with reduced toxicity and improved quality of life. Despite the fact the study was underpowered, these data suggest that a shorter duration leads to similar survival outcomes with better quality of life and thus might represent a new standard of care.

**Funding:**

Medical Research Council, Swedish Cancer Society, NETSCC, and Cancer Research UK.

## Introduction

Colorectal cancer is the fourth most common cancer worldwide, with 1 360 000 cases occurring annually, and is the fifth most common cause of death from cancer, causing 694 000 deaths.^1^ Postoperative adjuvant fluoropyrimidine chemotherapy was first shown to improve outcomes for patients with stage III colon cancer by Moertel and colleagues.^2^ The addition of oxaliplatin to a fluoropyrimidine chemotherapy backbone produced additional benefit,^3^, ^4^, ^5^ and oxaliplatin-containing chemotherapy is a recommended adjuvant treatment for stage III colon cancer.^6^, ^7^

**Research in context**

**Evidence before this study**

6 months of adjuvant oxaliplatin and fluoropyrimidine chemotherapy has been an option for patients with high-risk stage II and stage III colorectal cancer, as reflected in several international guidelines. Only one small study has investigated a 3 month duration of fluoropyrimidine adjuvant treatment. In view of the cumulative peripheral neuropathy that occurs with oxaliplatin, there was a question of whether a shorter duration of treatment would reduce toxicity without sacrificing efficacy. The SCOT study was designed to test for the non-inferiority of 3 months of treatment compared with the standard 6 month duration.

**Added value of this study**

Worldwide, six studies have addressed this research question, of which SCOT is the largest. Our results showed the non-inferiority of 3 months of adjuvant treatment compared with 6 months of treatment for the overall study population. Peripheral neuropathy was significantly worse in the 6 month group and reducing the treatment duration to 3 months more than halved the number of grade 3 or 4 peripheral neuropathy events reported. Moreover those patients who were most severely affected by peripheral neuropathy also had a significant reduction in quality of life.

**Implications of all the available evidence**

In view of the results of the SCOT study and the meta-analysis of all six worldwide studies conducted by the IDEA collaboration, 3 months of adjuvant chemotherapy will become the new global standard of adjuvant treatment for most patients who are suitable for treatment with CAPOX, particularly those patients with T1–3, N1 disease.

The major cumulative chronic toxic effect of oxaliplatin-containing chemotherapy is sensory peripheral neuropathy, which can be disabling. This effect is recognised to be dependent on dose and duration and can be long-lasting.^8^, ^9^ As most patients undergoing adjuvant chemotherapy are cured and will survive, long-term, irreversible peripheral neuropathy is a significant issue.

The current standard duration of adjuvant chemotherapy for colorectal cancer is 6 months. A shorter duration of adjuvant chemotherapy would be expected to result in less chronic peripheral neuropathy, but it has been hitherto unknown to what, if any, extent cutting duration would compromise its efficacy. The results of one study^10^ using fluoropyrimidine alone, which was not powered for non-inferiority, suggested that 3 months of infused fluoropyrimidine was similar to 6 months of bolus fluoropyrimidine treatment in terms of disease-free survival.^10^

The SCOT study was designed as a stand-alone international phase 3 non-inferiority study to investigate whether 3 months of oxaliplatin-based adjuvant chemotherapy for colorectal cancer is non-inferior to 6 months of treatment with the same regimen. Here we report the final efficacy results of disease-free survival and the toxicity and quality-of-life (QoL) results.

## Methods

### Study design and participants

The SCOT study is an international, randomised, non-blinded, non-inferiority, phase 3 trial comparing 6 months versus 3 months of oxaliplatin and fluoropyrimidine adjuvant chemotherapy in patients with high-risk stage II or stage III colorectal cancer. Patients were recruited from 244 centres in six countries (the UK, Denmark, Spain, Sweden, Australia, and New Zealand).

Patients were eligible if they were aged 18 years or older and had undergone a curative resection for stage III or high-risk stage II (defined as having one or more of T4 disease, tumour obstruction with or without perforation of the primary tumour preoperatively, fewer than ten lymph nodes harvested, poorly differentiated histology, perineural invasion, or extramural venous or lymphatic vascular invasion) adenocarcinoma of the colon or rectum. Patients were enrolled within 11 weeks of surgery and started treatment on their allocated study group within 2 weeks of randomisation. Other eligibility inclusion requirements included WHO performance status 0 or 1, adequate organ function, and life expectancy of greater than 5 years with reference to non-cancer related diseases. Patients had to have a normal CT scan of the chest, abdomen, and pelvis before study enrolment and carcinoembryonic antigen less than 1·2 times the local upper limit of normal (ULN) within 1 week before randomisation. Patients with rectal cancer had to have undergone total mesorectal excision surgery with negative resection margins (defined as >1 mm clearance). Exclusion criteria included chemotherapy (except chemotherapy administered with curative intent that was completed >5 years ago and from which there were no residual complications), previous long-course chemoradiotherapy (preoperative short-course radiotherapy alone was allowed), moderate or severe renal impairment (glomerular filtration rate or creatinine clearance <30 mL/min, as calculated with the Cockcroft-Gault equation), haemoglobin less than 9 g/dL, absolute neutrophil count less than 1·5 × 10^9^ cells per L, platelet count less than 100 × 10^9^ cells per L, and aspartate aminotransferase or alanine aminotransferase greater than 2·5 times the ULN. Other exclusion criteria were clinically significant cardiovascular disease, pregnancy or lactation or being of child-bearing potential and not using, or willing to use, medically approved contraception (postmenopausal women must have been amenorrhoeic for at least 12 months to be considered of non-childbearing potential), previous malignancy other than adequately treated in-situ carcinoma of the uterine cervix or basal or squamous cell carcinoma of the skin (unless there had been a disease-free interval of at least 5 years), and known or suspected dihydropyrimidine dehydrogenase deficiency.

The study was done in accordance with the Declaration of Helsinki and Good Clinical Practice guidelines. All aspects of the study received ethics approval from the National Research Ethics Service or its equivalent in the participating countries. This study was approved by the West Glasgow Research Ethics Committee (version 1.1 of the protocol) on Jan 21, 2008, and all subsequent amendments approved by the committee, where required (REC reference number 07/S0703/136). All participants provided written informed consent before enrolment. The full study details are available in the trial protocol, which is available online.

### Randomisation and masking

By use of a minimisation algorithm incorporating a random component, patients were centrally randomly assigned (1:1), to receive either 3 months or 6 months of treatment. The minimisation factors were centre, choice of regimen, sex, disease site (colon *vs* rectum), N stage (X, 0, 1, or 2) and T stage (X, 0, 1, 2, 3, or 4) and, if the patient was going to receive the CAPOX (capecitabine and oxaliplatin) regimen, the starting dose of capecitabine (from Feb 1, 2010).

Initially some participating centres were randomly allocated to randomise patients after completion of the first 3 months of treatment to either receive a further 3 months treatment or to stop treatment. This approach was stopped because of a poorer randomisation rate (ie, fewer patients randomised per month) compared with centres that randomised patients before the start of treatment.^11^

Participants were enrolled by authorised clinicians, after obtaining patient consent, by contacting the Cancer Research UK Clinical Trials Unit in Glasgow, UK, to check eligibility and request an allocation. At the end of the randomisation process, the computer randomisation system allocated every patient a unique identification number and determined their treatment duration. The study was open-label for patients, clinicians, and those doing the data analysis.

### Procedures

Patients were assigned to receive oxaliplatin-containing adjuvant treatment for either 3 months or 6 months. The chemotherapy regimen, which could be FOLFOX (bolus and infused fluorouracil with oxaliplatin) or CAPOX, was chosen by the physician and patient and was not randomised.

For patients receiving FOLFOX, treatment was given every 2 weeks with the intention of delivering six cycles to patients assigned 3 months of therapy and 12 cycles to patients assigned 6 months of therapy. Intravenous oxaliplatin 85 mg/m^2^ was given over 2 h on day one concurrently with L-folinic acid 175 mg or folinic acid (leucovorin) 350 mg. This was followed by an intravenous bolus injection of fluorouracil 400 mg/m^2^ over 5 min, then a continuous intravenous infusion of fluorouracil 2400 mg/m^2^ over 46 h. At the investigator's discretion, patients receiving FOLFOX who were older than 70 years could start on the bolus and continuous infusion of fluorouracil at 75% of the starting dose, if clinically indicated.

For patients receiving CAPOX, treatment was given every 3 weeks with an intention of delivering four cycles to patients assigned 3 months of therapy and eight cycles to patients assigned 6 months of therapy. Intravenous oxaliplatin 130 mg/m^2^ was given on day one over 2 h. Oral capecitabine 1000 mg/m^2^ was taken twice per day for the first 14 days of each cycle. Patients with a creatinine clearance of 30–50 mL/min had to start treatment with capecitabine at 75% of the full dose. Patients older than 70 years could be considered for treatment with capecitabine at 75% of the full dose, but the decision to reduce dose was left to the discretion of the investigator, depending on the fitness of the individual patient. If the clinician felt that any other patient required a-priori dose reduction because of any other comorbidity, that patient could be started on a minimum starting dose of oral capecitabine 800 mg/m^2^ twice per day.

Toxicity was assessed by the investigators after each cycle of chemotherapy treatment and graded by use of National Cancer Institute Common Terminology Criteria for Adverse Events (CTCAE) version three. If any grade 1 adverse event occurred as a result of chemotherapy, treatment was continued at the full dose. For all treatment-related adverse events of grade 2 or worse, treatment was withheld until recovery to grade 1, then restarted. For CAPOX, if more than one delay or a delay of at least 2 weeks occurred, capecitabine and oxaliplatin doses were reduced by 25% and, if further delays occurred due to myelotoxicity, further dose reductions were allowed at the investigators discretion. For FOLFOX, if more than one delay or a delay of at least 2 weeks occurred, doses of oxaliplatin and infused fluorouracil were maintained, but the bolus fluorouracil was omitted and, if further delays occurred due to myelotoxicity, the oxaliplatin and infusional fluorouracil doses were reduced by 25%. Also for FOLFOX, if after the first cycle neutrophils were less than 1·0 × 10^9^ cells per L, the bolus fluorouracil was omitted and the oxaliplatin and infused fluorouracil doses were reduced by 25%. Wherever possible for toxic effects, the oxaliplatin dose was reduced rather than discontinued. If oxaliplatin was discontinued, the fluoropyrimidine was continued if possible. Patients were followed up for 8 years with full blood count, urea and electrolyte, liver function, and carcinoembryonic antigen tested at months 9, 12, 18, 24, and 36, then annually. CT of the chest, abdomen, and pelvis was done at months 6, 12, 18, 24 and 36.

QoL was assessed with the European Organisation for Research and Treatment of Cancer (EORTC) QLQ-C30 and QLQ-CR29 and EQ-5D-3L (visual analogue scale and health index), with UK value sets.^12^ Neuropathy was assessed with the FACT/GOG-Ntx4 questionnaire.

The QoL questionnaires were administered at baseline and before each treatment cycle. Additionally, QoL was assessed each month in the 3 month group for the first 3 months after treatment. Subsequent assessments were made at months 9 and 12 for the EORTC questionnaires and months 9, 12, 18, and 24, then annually for EQ-5D-3L.

Neuropathy assessments with FACT/GOG-Ntx4 were initially completed up to 12 months, as were the EORTC questionnaires in patients who underwent randomisation before Feb 16, 2011, from sites that opted to participate in this substudy. An amendment introduced in version 3.0 of the protocol on March 20, 2012, extended the requirement for the neuropathy questionnaire to be completed for patients who completed it at baseline to the follow-up visits at months 18 and 24. A further amendment (version 4.0 on Dec 20, 2012) extended the requirement for the neuropathy questionnaire to be completed to every follow-up visit (to a maximum of 8 years) for all new patients and existing patients already participating in the substudy. These amendments were made as a result of recommendations by the independent data monitoring committee.

### Outcomes

The primary study endpoint was disease-free survival, defined as the time from randomisation (or trial registration for those randomised after 3 months of therapy) to relapse, development of a new colorectal cancer, or death from any cause. Secondary endpoints were overall survival, safety, QoL, and cost-effectiveness, which will be reported separately. Overall survival was defined as the time from randomisation (registration for those randomised at 3 months) to death from any cause. At selected centres, patients were entered into a translational substudy, the TransSCOT study and had tissue and blood samples collected.

### Statistical analysis

In the MOSAIC trial, 3 year disease-free survival in the FOLFOX group was 78% as compared with 73% with fluorouracil and leucovorin.^3^ To conclude non-inferiority for the 3 month group, we would wish to be confident that at least half of this benefit was retained.

SCOT was designed as a non-inferiority trial aiming to reliably determine whether there was less than a 2·5% fall in 3 year disease-free survival for patients in the 3 month treatment group (from 78% in the 6 month group), which corresponds to excluding a hazard ratio (HR) greater than 1·13 with 90% power at the 2·5% one-sided level of significance. Assuming that the study would recruit over a period of 5 years with a subsequent minimum follow-up of 2 years, this design required 8600 patients to undergo randomisation and 2750 events (relapses, deaths, or new colorectal cancers) to be observed. To allow for losses to follow-up, our target recruitment was 9500 patients.

From the outset, we recognised that reliable conclusions based on safety and QoL data would not require information on all 9500 patients. For safety outcomes, the plan was that 700 patients (350 in each group) would be sufficient to detect (with 80% power and two-sided significance level of 5%) a halving in the proportion of patients with grade 3 or 4 toxic effects from 12% to 6% (12% being the rate at which grade 3 or 4 paraesthesia—the most frequent non-haematological grade 3 or 4 toxic effect—occurred in the oxaliplatin group in the MOSAIC trial).^3^ This same sample size would allow small changes in global QoL to be detected (assuming a difference of magnitude of 7·53^13^ and a standard deviation of 23·4)^14^ with 95% power at the 1% significance level. This more stringent level of significance was used to allow for multiple testing across the various QoL scales.

We collected information on these toxicity and QoL endpoints from all patients recruited until the number required was exceeded and the decision to stop was endorsed by the independent data monitoring committee and trial steering committee. An administrative delay in notifying the sites about the need to end the collection of detailed toxicity data resulted in data being collected from 868 patients. The data monitoring committee had access to summary plots of EORTC QoL data, EQ-5D health status, and FACT/GOG-Ntx4 neuropathy data by study group and study timepoints and following a committee meeting on May 28, 2010 (based on 1047 randomised patients), recommended that the collection of QoL and FACT/GOG-Ntx4 data be continued because they were concerned that the amount of missing data might undermine the comparisons at later timepoints if the original sample size estimates were used. They also recommended that the collection of FACT/GOG-Ntx4 data should be extended beyond 12 months for new patients and, where possible, for patients already on the study. At their next meeting on Nov 23, 2010, the data monitoring committee recommended that the collection of QoL and FACT/GOG-Ntx4 data should stop once 1800 patients had been recruited. Delays in the amendment of the protocol to implement the collection of the FACT/GOG-Ntx4 beyond 12 months led to patient recruitment beyond this recommendation to ensure sufficient numbers of patients at these later timepoints. These recommendations were subsequently endorsed by the trial steering committee. These extensions to data collection were made to compensate for missing data and no formal power calculations were made for these changes.

The efficacy analyses of disease-free survival and overall survival included all randomly assigned patients (intention-to-treat population) as far as possible. Kaplan-Meier techniques were used to plot both disease-free survival and overall survival. The analysis of treatment delivery and safety was based on patients who started study treatment.

The comparison of disease-free survival and overall survival between the treatment groups was based on a Cox regression model incorporating the minimisation factors as covariates. The HR associated with the treatment groups was derived from this model along with the associated 95% CI. The p value for testing the null hypothesis that the HR of 3 months versus 6 months was greater than or equal to 1·13 was derived from this model by comparing the log-likelihood of the fitted model with the log-likelihood of a model where the HR between the groups is set to 1·13 by use of a likelihood ratio test. The proportional hazards assumption implicit in these analyses was examined graphically via a log-minus log plot of the survival function against log time and via a test of the interaction between the treatment group and time (logged) obtained from a Cox model incorporating an appropriate time-varying covariate.

The components of the forest plot (estimated HR for the comparison between groups and associated 95% CI) were derived from a Cox model that included separate terms for the effect of duration within each category of the relevant stratification factor or other factor being examined. The p value for heterogeneity was derived from a comparison of the log-likelihoods of a model with separate terms for the effect of duration within each category with the model with a single overall term. The aim of this analysis was to establish whether the impact of treatment duration varied across important patient subgroups.

Multiple imputation analysis^15^ was used to fill in the missing data and questionnaires in the QoL and neuropathy scales. Five multiple imputation sets were produced for each QoL or neuropathy scale and the area under the curve (AUC)^16^ was calculated with the imputed data. This was prespecified in the statistical analysis plan. This AUC was then adjusted by dividing by the follow-up period and subtracting the baseline value for each patient to produce a standardised adjusted AUC. The standardised adjusted AUC was calculated for the five imputed datasets and compared between the randomised treatment groups via a generalised linear model (with study group as an independent factor and study minimisation factors as covariates). The test statistics associated with study group from each of the five imputations were finally combined to provide an overall p value that takes into account the extent of the missing data. To allow for the number of scales being examined, an adjustment for multiple comparisons (separately for the EORTC and EQ-5D questionnaires) was made with the sharpened Hochberg procedure.^17^ The p value threshold for statistical significance was 5% after adjustment. Comparisons of these scales at individual timepoints also made use of multiple imputation and generalised linear models.

The Mann-Whitney *U* test was used for the comparison of ordered categorical variables for toxicity grade. Fisher's exact test was used to compare the incidence of grade 3–5 toxic effects and the odds ratio and associated confidence interval for the incidence of grade 3–5 toxicity was estimated using logistic regression.

The study data were reviewed by the data monitoring committee approximately once per year to assess safety and efficacy issues from an ethical viewpoint. Conditional power methods^18^ were used to aid the committee in reaching decisions about study continuation, but no formal stopping rules were set. The conditional power for disease-free survival was presented at the fifth (June 26, 2012), sixth (Jan 7, 2013), and seventh (Oct 2, 2013) meetings of the data monitoring committee. The data monitoring committee requested the analysis because of apparent differences in disease-free survival curves. The conditional power results were discussed by the data monitoring committee in the context of the limited follow-up on patients and the available survival data. The data monitoring committee also had access to interim disease-free survival results from an Italian study TOSCA (NCT00646607), which was addressing the same question and had more mature data at the time. The data monitoring committee concluded that no action was required taking all information into account.

All statistical analyses were done with SPSS version 22 and R version 3.1.2. This trial is registered with ISRCTN, number ISRCTN59757862, and follow-up is continuing.

### Role of the funding source

The funders of the study had no role in study design, data collection, data analysis, data interpretation, or writing of the report. All authors had full access to all the raw data and the corresponding author had full access to all the data in the study and had final responsibility for the decision to submit for publication.

## Results

**Figure 1:**
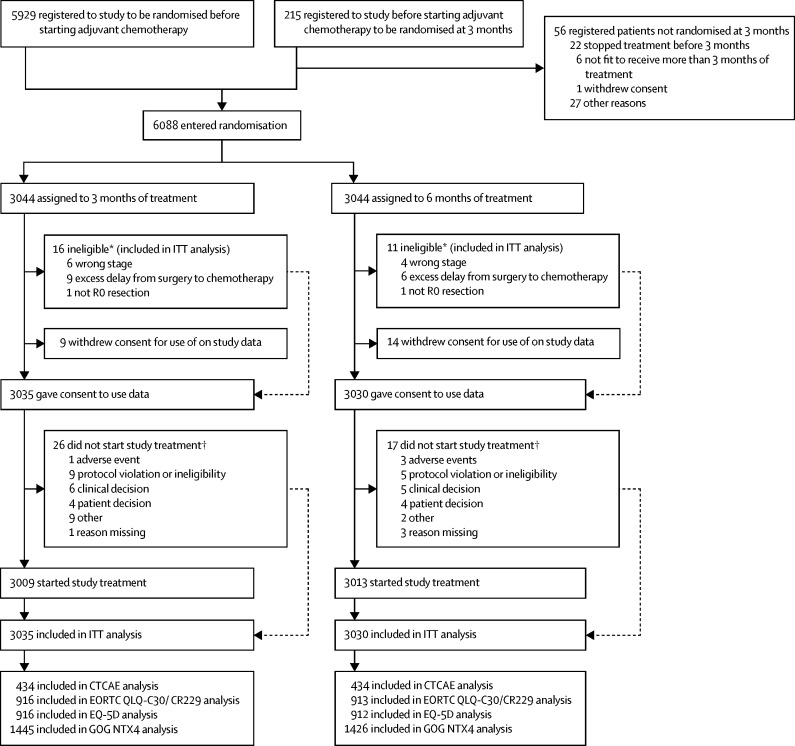
Trial profile ITT=intention to treat. CTCAE=Common Terminology Criteria for Adverse Events. *Based on retrospective review. †Patients could have more than one reason.

Between March 27, 2008, and Nov 29, 2013, 6088 patients underwent randomisation (figure 1) at 244 centres, including 5244 patients at 164 centres in the UK, 197 patients at 32 centres in Australia, 237 patients at 19 centres in Spain, 83 patients at 14 centres in Sweden, 311 patients at ten centres in Denmark, and 16 patients at five centres in New Zealand. The study did not meet its recruitment target of 9500 patients because accrual was not as rapid as originally forecast and recruitment needed to stop to allow sufficiently complete follow-up on current patients within the available funding. By increasing the recruitment period by 6 months and extending minimum follow-up to 3 years (88% patients were followed up for 3 year disease-free survival by the reverse Kaplan-Meier method, allowing for a 2 month deviation from the assessment time), 1482 disease-free survival events were observed, giving the study 66% power rather than the originally planned 90% power for rejecting the null hypothesis. Results from previous adjuvant studies have shown that most disease-free survival events occur within the first 3 years of starting treatment.^3^, ^19^ The analysis time was prespecified in the protocol and therefore no bias would be introduced as might be the case if we had allowed the timing of the analysis to be guided by the data at hand.

The data cutoff for this analysis was Dec 1, 2016. The median follow-up time in both groups was 37 months (IQR 36–49), which we calculated by the reverse Kaplan-Meier approach. 787 patients had died at the time of analysis.

The baseline demographics, stage, and site of disease for each group were balanced across the trial population (table 1). The corresponding information for the patients with data on CTCAE-graded adverse events and EQ-5D, EORTC QLQ-C30/CR229, and FACT/GOG-Ntx4 are shown in the appendix (pp 6–9). Patients were also well balanced across the treatment groups within these subgroups, with a similar overall distribution of patient characteristics to the main study population. The largest difference between the overall population and the subgroups assessed for safety and QoL was a 9% increase in the incidence of patients with performance status 1 in the CTCAE safety cohort; all other differences in individual characteristics were within 5%.

**Table 1:** Baseline patient characteristics recorded at randomisation by study group

	**3 months of treatment (n=3044)**	**6 months of treatment (n=3044)**
**Sex**		
Female	1201 (39%)	1200 (39%)
Male	1843 (61%)	1844 (61%)
**Age (years)**		
Median	65 (58–70)	65 (58–70)
**Performance status at randomisation**		
0	2190 (72%)	2144 (70%)
1	854 (28%)	900 (30%)
**Disease site**		
Colon	2492 (82%)	2495 (82%)
Rectum	552 (18%)	549 (18%)
**T stage**		
0	1 (<1%)	3 (<1%)
1	92 (3%)	95 (3%)
2	284 (9%)	283 (9%)
3	1749 (57%)	1748 (57%)
4	917 (30%)	915 (30%)
X	1 (<1%)	0
**N stage**		
0	559 (18%)	557 (18%)
1	1731 (57%)	1732 (57%)
2	754 (25%)	755 (25%)
**Planned treatment**		
FOLFOX	993 (33%)	988 (32%)
CAPOX	2051 (67%)	2056 (68%)
**Starting dose of capecitabine if CAPOX planned**		
750 mg/m^2^	348 (19%)	349 (19%)
800 mg/m^2^	72 (4%)	78 (4%)
1000 mg/m^2^	1369 (77%)	1370 (76%)
Number of patients with data available	1789	1797
**High risk stage II**		
No	2493 (82%)	2499 (82%)
Yes	551 (18%)	545 (18%)
**Randomisation timepoint**		
Baseline	2964 (97%)	2965 (97%)
3 months	80 (3%)	79 (3%)

Data are n (%) or (IQR) unless noted otherwise.

**Figure 2:**
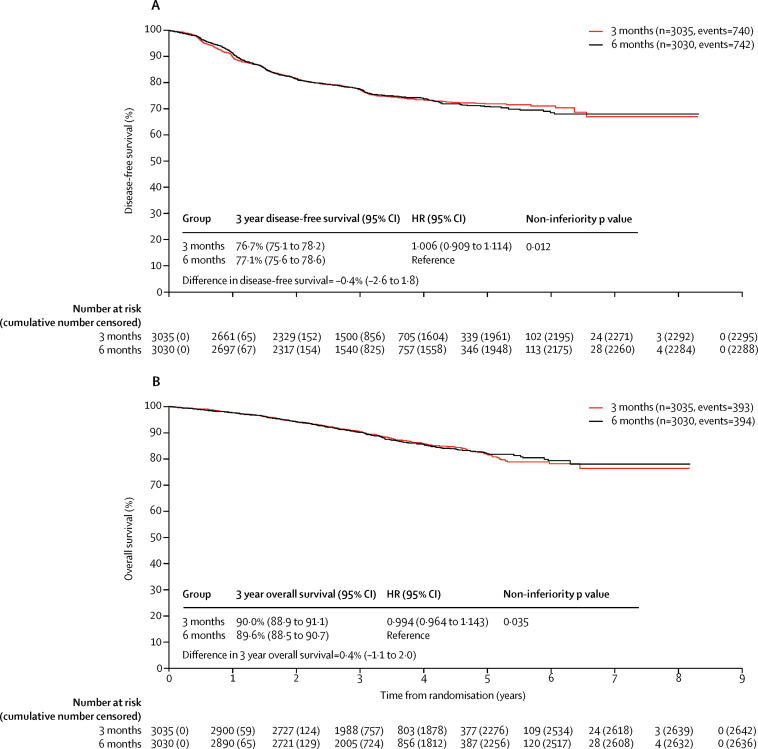
Disease-free survival (A) and overall survival (B) by study group HR=hazard ratio.

At the time of the disease-free survival analysis there had been 740 events in the 3 month group and 742 events in the 6 month group. 3 year disease-free survival was 76·7% (95% CI 75·1–78·2) in the 3 month group and 77·1% (75·6–78·6) in the 6 month group, giving an HR of 1·006 (0·909–1·114, p=0·012) which met the criteria for non-inferiority (figure 2).

3 year overall survival is shown in figure 2. 393 patients died in the 3 month group versus 394 in the 6 month group. 16 deaths in each group were related to the protocol treatment. The appendix contains a breakdown of causes of death (p 10) and further details of deaths related to protocol treatment (p 11).

In a prespecified sensitivity analysis, we also examined the difference between the study groups according to the actual duration of treatment on the two randomised groups (appendix p 1). None of these analyses showed non-inferiority. For eligible patients who received the actual assigned study duration of 3 and 6 months, the observed HR was 1·158 (95% CI 1·018–1·317, p=0·641).

**Figure 3:**
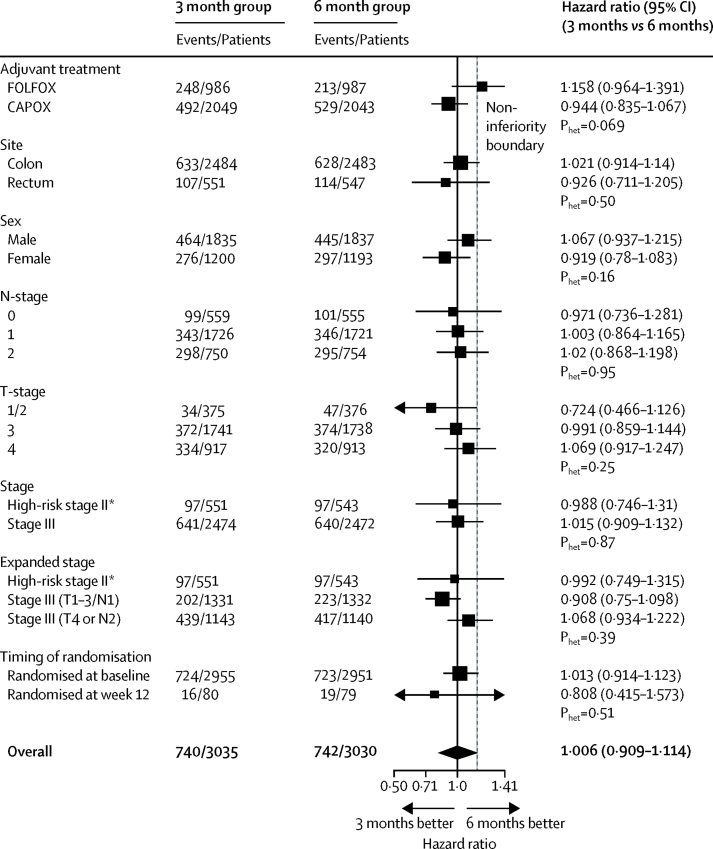
Disease-free survival and heterogeneity in subgroups by minimisation variables Categories are listed as recorded at randomisation; ten patients in the 3 month group and 15 patients in the 6 month group could not be allocated to high risk stage II or stage III based on T/N data recorded at randomisation. *These estimates differ slightly because the underlying multivariable Cox model on which they are based includes parameters for other minimisation variables, as well as those factors relating to stage; the increased flexibility in the expanded stage model allows the influence of these parameters on the high risk stage II estimates to modify.

**Figure 4:**
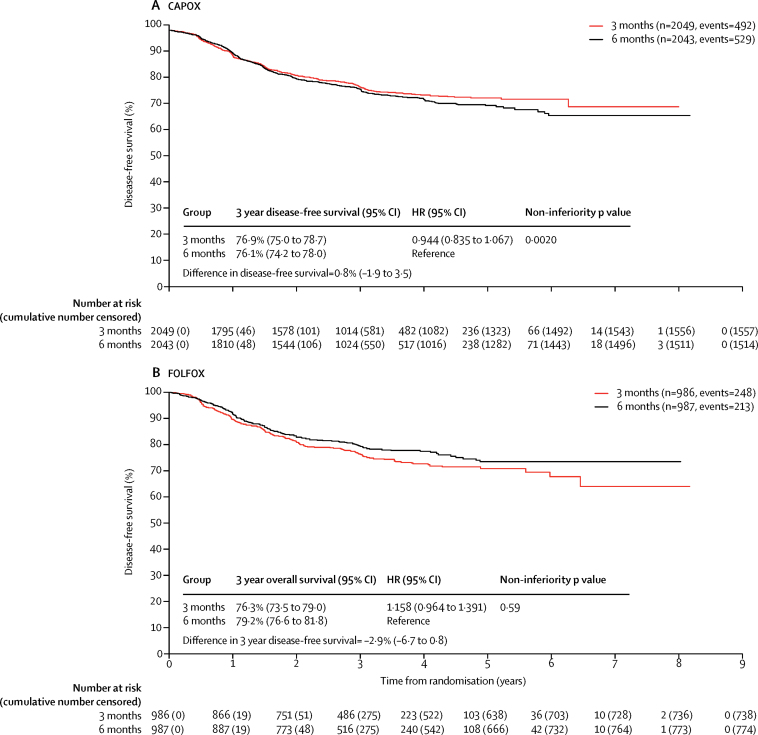
Disease-free survival by study group and selected adjuvant regimen HR=hazard ratio.

Forest plots by stratification factors and randomisation timepoint are shown in figure 3. The most marked heterogeneity was for the dependence of the duration effect on the initial choice of regimen (p=0·069), so we did a post-hoc analysis of disease-free survival for the two durations of therapy for CAPOX and FOLFOX regimens (figure 4). For those patients receiving CAPOX the test for non-inferiority in this post-hoc analysis was statistically significant (p=0·0020).

Among patients with available chemotherapy duration data, 2521 (83%) of 3024 of patients assigned to 3 months of treatment actually received 3 months of treatment, including 845 (86%) of 980 receiving FOLFOX and 1676 (82%) of the 2044 receiving CAPOX. 1773 (59%) of 3013 patients assigned to receive 6 months of treatment actually received 6 months of treatment, which was similar for those receiving FOLFOX (585 [59%] of 985) and CAPOX (1188 [59%] of 2028). 209 (7%) of 3013 patients assigned to receive 6 months of treatment stopped treatment at 3 months. Overall, 842 (14%) of 6037 patients stopped treatment before 3 months and this was roughly evenly split between those assigned to receive 3 months (425 [14%] of 3024) and 6 months (417 [14%] of 3013) of treatment.

244 patients in the 3 month group and 576 patients in the 6 month group cited adverse events as the reason for stopping treatment early. The most commonly cited individual adverse event causing treatment to be stopped in the 3 month group was diarrhoea (90 patients). In the 6 month group, both diarrhoea (150 patients) and peripheral neuropathy (156 patients) were commonly cited as reasons for stopping.

**Figure 5:**
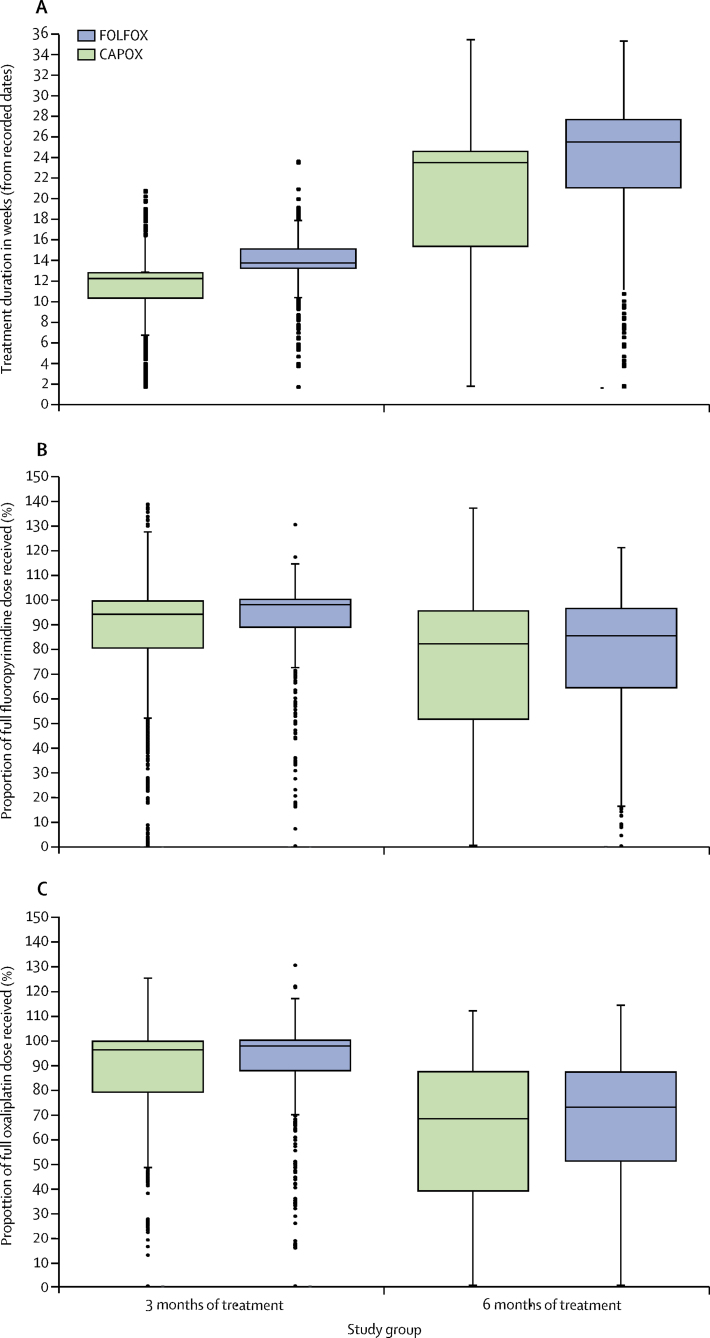
Treatment delivery by selected adjuvant regimen Box and whisker plots indicate median and IQR (boxes) and range (whiskers). Dots represent outliers.

Figure 5 shows the dose delivery for fluoropyrimidine and oxaliplatin. The median percentage of full fluoropyrimidine dose delivered was 95·3% (IQR 83·1–99·8) in the 3 month group and 83·2% (56·7–95·7) in the 6 month group. The corresponding figures for oxaliplatin dose delivery were 96·6% (82·3–99·7) in the 3 month group and 70·2% (44·3–87·1) in the 6 month group. These figures were similar irrespective of the fluoropyrimidine backbone. The number of patients who had recorded fluorouracil or prescribed capecitabine dose reductions in the 3 month group were 788 (26%) of 3009 compared with 1286 (43%) of 3013 in the 6 month group. For oxaliplatin, the corresponding figures were 906 (30%) of 3009 and 1869 (62%) of 3013.

**Figure 6:**
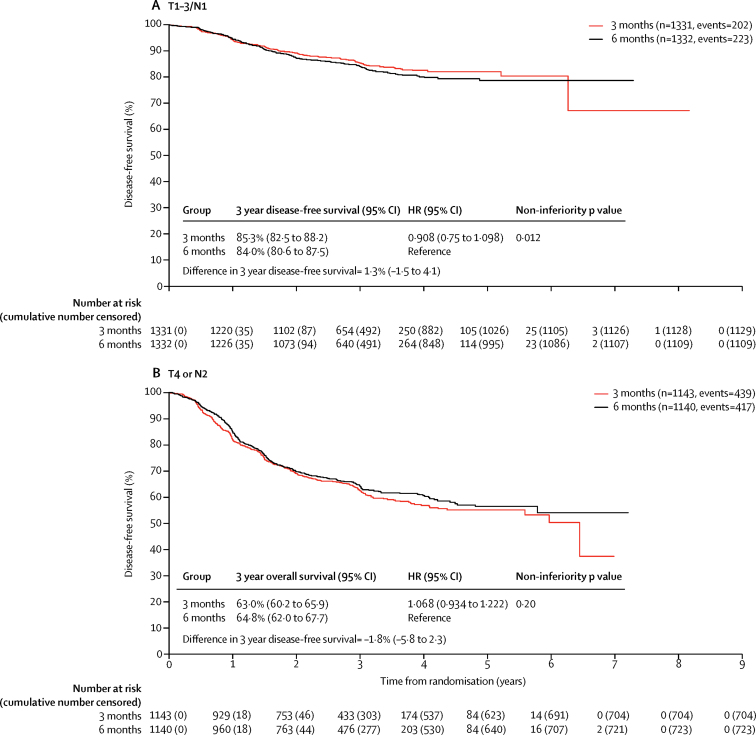
Disease-free survival by study group and stage III risk group HR=hazard ratio.

Given previous trial data,^20^ it is known that there is a marked difference in risk of relapse between patients with N1 colorectal cancer compared with those with N2 pathology, so we did a post-hoc analysis comparing T1–3, N1 colorectal cancer against T4 and N2 pathology (figure 6), and analysed these different prognostic groups according to the chemotherapy regimen they received (FOLFOX or CAPOX; appendix p 5). For those patients with T1-3/N1 disease the test for non-inferiority in this post-hoc analysis was statistically significant (p=0·012).

Safety was assessed by the investigators in 868 patients, 434 (50%) in each group. Sensory neuropathy, diarrhoea, neutropenia, fatigue, pain, nausea, and hand-foot syndrome were the most common grade 3–5 adverse events (table 2). The frequency of grade 3–5 diarrhoea (p=0·033), neutropenia (p=0·031), pain (p=0·014), hand-foot syndrome (p=0·031), and sensory neuropathy (p<0·0001) was significantly higher in the 6 month group than in the 3 month group. Diarrhoea and hand-foot syndrome were more frequent in patients receiving CAPOX and neutropenia was more frequent in patients receiving FOLFOX (appendix p 5). The only side-effect for which the percentage of patients with adverse events of grade 2 or worse differed by more than 10% between the two treatment groups was sensory neuropathy (237 [58%] of 409 patients with data in the 6 month group *vs* 103 [25%] of 420 patients with data in the 3 month group). Overall, grade 3–5 adverse events were reported by 150 (36%) of 420 patients in the 3 month group and 243 (59%) of 409 patients in the 6 month group (p<0·0001). Serious adverse reactions to treatment were recorded for all patients who started study treatment. In the 3 month group, 421 patients had serious adverse reactions (492 individual reports) compared with 511 patients (606 individual reports) in the 6 month group. A breakdown of serious adverse reactions by body system is shown in the appendix (p 12). Gastrointestinal serious adverse reactions were the most common and occurred at similar frequencies in both groups. Adverse events of grade 3 or worse with an overall frequency less than 10% are shown in the appendix (p 13).

**Table 2:** Maximum grade of adverse event during treatment

	**3 months of treatment (n=434)**						**6 months of treatment (n=434)**						**Comparisons of 6 months *vs* 3 months**		
	0	1–2	3	4	5	Patients with missing data	0	1–2	3	4	5	Patients with missing data	Ordered categories (p value)	Proportion of patients with grade 3–5 adverse event (p value)	Frequency of grade 3–5 event (odds ratio [95% CI])
Alopecia	345 (83%)	69 (17%)	0	0	0	20	309 (76%)	97 (24%)	0	0	0	28	0·0094	..	Not estimable
Anaemia	270 (65%)	143 (34%)	2 (<1%)	2 (<1%)	0	17	212 (52%)	190 (47%)	3 (<1%)	1 (<1%)	0	28	0·00013	1·00	1·027 (0·255–4·136)
Anorexia	312 (76%)	92 (22%)	7 (2%)	0	0	23	262 (65%)	140 (35%)	3 (<1%)	0	0	29	0·00043	0·34	0·431 (0·111–1·677)
Constipation	289 (70%)	122 (29%)	2 (<1%)	1 (<1%)	0	20	268 (66%)	135 (33%)	3 (<1%)	0	0	28	0·28	1·00	1·010 (0·205–5·083)
Diarrhoea	128 (31%)	243 (58%)	44 (11%)	2 (<1%)	0	17	99 (24%)	241 (59%)	63 (16%)	2 (<1%)	1 (<1%)	28	0·0079	0·033	1·566 (1·045–2·345)
Fatigue	58 (14%)	320 (77%)	35 (8%)	2 (<1%)	0	19	41 (10%)	333 (82%)	32 (8%)	0	0	28	0·022	0·62	0·874 (0·533–1·433)
Hand-foot syndrome	277 (67%)	129 (31%)	8 (2%)	0	0	20	218 (54%)	169 (42%)	18 (4%)	1 (<1%)	0	28	<0·0001	0·031	2·492 (1·078–5·758)
Mucositis (clinical examination)	355 (86%)	56 (14%)	2 (<1%)	0	0	21	320 (79%)	83 (20%)	1 (<1%)	0	1 (<1%)	29	0·013	1·00	1·020 (0·143–7·275)
Mucositis (functional or symptomatic)	283 (68%)	127 (31%)	4 (1%)	0	0	20	242 (60%)	159 (39%)	4 (1%)	0	1 (<1%)	28	0·0066	0·75	1·278 (0·341–4·794)
Nausea	147 (35%)	249 (60%)	20 (5%)	0	0	18	120 (30%)	277 (68%)	9 (2%)	0	0	28	0·26	0·057	0·449 (0·202–0·998)
Sensory neuropathy	37 (9%)	365 (87%)	18 (4%)	0	0	14	28 (7%)	314 (77%)	65 (16%)	2 (<1%)	0	25	<0·0001	<0·0001	4·375 (2·550–7·508)
Neutropenia	287 (69%)	90 (22%)	23 (6%)	16 (4%)	0	18	221 (54%)	127 (31%)	43 (11%)	14 (3%)	1 (<1%)	28	<0·0001	0·031	1·611 (1·046–2·480)
Pain, other	311 (74%)	99 (24%)	10 (2%)	0	0	14	278 (68%)	107 (26%)	24 (6%)	0	0	25	0·026	0·014	2·556 (1·206–5·415)
Rash	359 (87%)	52 (13%)	2 (<1%)	0	0	21	320 (79%)	84 (21%)	1 (<1%)	0	0	29	0·00061	1·00	0·509 (0·046–5·632)
Taste alteration	231 (56%)	180 (44%)	1 (<1%)	0	0	22	179 (44%)	222 (55%)	2 (<1%)	0	0	31	0·0021	0·57	2·050 (0·185–22·696)
Thrombocytopenia	290 (70%)	117 (28%)	5 (1%)	4 (1%)	0	18	253 (62%)	145 (36%)	5 (1%)	2 (<1%)	1 (<1%)	28	0·020	1·00	0·909 (0·347–2·380)
Vomiting	304 (73%)	98 (24%)	14 (3%)	0	0	18	270 (67%)	126 (31%)	10 (2%)	0	0	28	0·056	0·54	0·725 (0·318–1·652)
Watery eye	339 (83%)	71 (17%)	0	0	0	24	310 (77%)	92 (23%)	2 (<1%)	0	0	30	0·028	0·25	Not estimable

Data are n (%) or n unless specified otherwise. No grade 5 events occurred in the 3 month group. Adverse events with an incidence ≥10% are shown. Percentages are calculated from the total number of patients minus the number of patients with missing data in each row. Odds ratios are calculated from logistic regression as 6 months *vs* 3 months. Adverse events were graded in accordance with the National Cancer Institute Common Terminology Criteria for Adverse Events.

**Figure 7:**
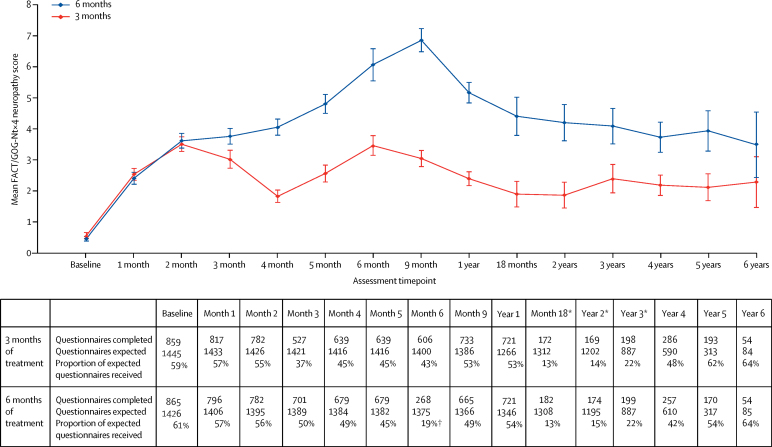
Peripheral neuropathy by study group Patients reported peripheral neuropathy with the GOG Ntx4 questionnaire. Although results were available beyond year 6, they have been omitted because of small numbers and resultant wide confidence intervals. Error bars show 95% CIs. *The low completion rates at these timepoints reflect the fact that, initially, neurotoxicity data were only collected up to 12 months and there was a delay before an amendment extended the collection to the whole study period. †Low return rate because patients were assessed at the start of last cycle rather than 6 months (which corresponds to end of cycle).

In addition to the assessment using the CTCAE during treatment, peripheral neuropathy was also assessed using a patient recorded outcome FACT/GOG-Ntx4 questionnaire. FACT/GOG-Ntx4 data were available from 2871 patients. We compared the results from the FACT/GOG-Ntx4 questionnaire for patients receiving 3 months of chemotherapy with those receiving 6 months of chemotherapy (figure 7). The neuropathy standardised adjusted AUC differed significantly between the two study groups (p<0·0001), with clear differences appearing at month 4 and persisting until at least 5 years (p<0·0001). Peak neuropathy occurred at 9 months in the 6 month group and at 6 months in the 3 month group.

1829 patients provided data for EORTC QLQ-C30 and QLQ-CR29. All the functional and symptom scales in EORTC QLQ-C30 showed significant differences favouring the 3 month group (ie, fewer side-effects and better function) in terms of standardised adjusted AUC (p<0·0001 for all functional scales adjusted for multiple testing; appendix p 15). For the EORTC QLQ-CR29 questionnaire, significant differences were detected in the body image (p=0·037), dry mouth (p<0·0001), hair loss (p=0·035), and taste scales (p<0·0001; all p values adjusted for multiple testing; appendix p 15).

The global health status and the functional and symptom scales that significantly differed between the groups matched each other during the first 3 months of treatment, then separated between months 3 to 6 as function improved or symptoms decreased in patients who had stopped treatment at 3 months (appendix pp 2, 15). The maximum difference in means was generally seen at 6 months and, in accordance with the categories of Osoba and colleagues,^13^ these mean differences in functional and global health status scales represented “moderate” changes (values between 10–20) for global health status, role functioning, and social function and “a little” change (values between 5–10) for physical functioning, emotional functioning, and cognitive functioning. Thereafter, the mean values converged during months 9 and 12 after patients in the 6 month group had stopped treatment. The largest difference in means at 12 months was 3·79 (SE 1·06) for role functioning (appendix p 15). The graph for global health status is shown in the appendix (p 2).

1828 patients provided data for EQ-5D-3L. The standardised adjusted AUCs differed significantly for both the EQ-5D self-rated visual analogue scale (p=0·00081) and the EQ-5D-3L health index (p=0·00081; appendix p 15). Plotting the EQ-5D scales showed that the differences between the two groups of the study were restricted to months 4, 5, and 6, the period when patients in the 6 month group were still receiving treatment and those in the 3 month group had stopped. The maximum difference in means was at 6 months and these differences were consistent with clinically important differences (appendix p 15).^21^ This pattern was also noticeable for the EQ-5D self-rated visual analogue scale; patients in the 6 month group had significantly lower QoL than those in the 3 month group during months 4, 5, and 6, but after this point, there were no overall clinically important differences up to the 6 year follow-up (appendix p 3).

To investigate the potential effects of missing data on the results from the FACT/GOG-Ntx4, EORTC, and EQ-5D assessments, the reasons for missing questionnaires were recorded (appendix p 16). The reasons were broadly similar between the groups and were mainly related to errors of various kinds. Similarly, an analysis of missingness according to baseline factors indicated that the patients who completed the questionnaires were representative of the patients who participated in this aspect of the study, with any associations between a questionnaire being missing and baseline characteristics being small (appendix p 17). Finally, we did a sensitivity analysis to compare the primary imputed results with the results based on observed data only or the imputed results for patients who completed baseline questionnaires only. The results of these analyses were all very similar to the findings of the main study (appendix p 18).

We did an exploratory analysis to examine the differences in QoL scales between patients whose worst responses to the questions on the GOGNtx4 questionnaire about whether they had numbness, tingling, or discomfort in their hands or feet were “Quite a bit” or “Very much” and those whose worst responses were “Somewhat“, “A little bit”, or “Not at all” (appendix p 4). This analysis identified statistically and clinically significant poorer QoL between these patient groups at 1 year, 3 years, and 5 years, apart from EQ-5D visual analogue scale at 1 year, for which the difference was not clinically significant. The proportion of patients recording neuropathy symptoms as being present “Quite a bit” or “Very much” was significantly higher in the 6 month group than in the 3 month group at each timepoint (248 [34%] of 721 *vs* 99 [14%] of 721, p<0·0001 at 1 year; 63 [32%] of 199 *vs* 30 [15%] of 198, p<0·0001 at 3 years; 49 [29%] of 170 *vs* 30 [16%] of 193, p=0·0032 at 5 years).

## Discussion

Our results show the non-inferiority of 3 months of adjuvant oxaliplatin-containing chemotherapy treatment compared with 6 months of treatment for patients with high-risk stage II and stage III cancer of the colon and rectum. The shorter treatment duration was also associated with reduced toxicity and less deterioration in QoL. Because this study recruited 6088 patients with conventionally defined high-risk stage II and stage III colorectal cancer from a large number of centres and countries and made use of standard chemotherapy regimens, the study findings are applicable to a typical patient with colorectal cancer needing adjuvant chemotherapy treatment. The non-inferiority boundary was set to exclude a maximum 2·5% fall in 3 year disease-free survival in patients in the 3 month group compared with those in the 6 month group, which—as estimated on the basis of results from previous trials—would yield a predicted 3 year disease-free survival of 78%. This threshold of 2·5% was chosen because it was half the difference seen in 3 year disease-free survival between patients in the oxaliplatin-containing group and those in the fluoropyrimidine only group in the MOSAIC study. It was felt by clinicians commonly treating colorectal cancer that this small difference would be an acceptable payoff to bring about a significant reduction in long-term neuropathy and potential improvement in QoL to patients who achieve a long-term cure. This difference corresponds to an HR of 1·13, which we aimed to detect with 90% power at the 2·5% one-sided level of statistical significance.

Because 6088 patients were recruited and 1482 events were recorded (rather than our initial target of 9500 patients with 2750 events), the power was reduced to 66%. However, we were still able to show the non-inferiority of 3 months of treatment compared with 6 months of treatment (p=0·012) across the trial population as a whole (stage III and high risk stage II disease; colon and rectal cancer), with 3 year disease-free survival of 77·1% (95% CI 75·6 to 78·6) for patients in the 6 month group and 76·7% (75·1 to 78·2) for those in the 3 month group (HR 1·006, 0·909 to 1·114). The absolute loss in 3 year disease-free survival with 3 months of treatment was 0·4% (–1·8 to 2·6). Patients in the 6 month group had similar 3 year disease-free survival to that seen with 6 months of oxaliplatin-containing adjuvant chemotherapy in the MOSAIC study (78·2%) and NSABP C07 studies (76·1%), suggesting that the outcome observed in our control group was similar to that previously seen.^3^, ^4^ However, it is important to note that in the SCOT trial, only 1096 (18%) of 6088 patients had high-risk stage II disease, whereas in MOSAIC and NSABP-C07, 40% and 30% of patients had all-risk stage II disease. Our definition of high risk-stage II disease was the same as that used in MOSAIC. At the time SCOT was initiated, less data were available on adjuvant chemotherapy for rectal cancer because many adjuvant studies have excluded these patients, and patients with rectal cancer were eligible to participate if they had received no preoperative chemotherapy (short-course radiotherapy alone was allowed) and had undergone total mesorectal excision with an R0 resection. The only adjuvant chemotherapy these patients received was within this study and randomised for the duration of treatment.

We did comparisons of disease-free survival based on the actual duration of treatment received in the study groups, as well as the primary intention-to-treat analyses. These subanalyses did not show non-inferiority, but are inherently biased by the differential exclusion of patients not able to receive prolonged therapy because of the different target treatment durations in the study groups. In a setting such as this where differential compliance is intrinsic to the treatments being compared, our view is that the intention-to-treat analysis is a more accurate reflection of the impact of the intervention on actual clinical practice.

In terms of our analyses of smaller subgroups, we have not been able to draw solid conclusions about the effect of treatment duration on specific patient populations such as those with high-risk stage II disease or patients with rectal cancer because the numbers in these subgroups were relatively small and few events occurred. However, the Forest plots did not show any differences in the effect of the duration of adjuvant chemotherapy between patients with high-risk stage 2 disease and those with stage III disease and between those with rectal cancer and those with colon cancer. Therefore, there is no reason to consider that these subpopulations should be treated differently in clinical practice to the trial population as a whole. We can only apply the SCOT results to patients being considered for doublet chemotherapy as tested within the study. In clinical practice in many parts of the world, a substantial proportion of patients with high-risk stage II disease or stage III disease (especially if older than 70 years of age) receive only single-agent fluoropyrimidine for 6 months because the addition of oxaliplatin to fluoropyrimidine has not been shown to improve survival in these patients.^22^

Other factors to consider in terms of prognosis and prediction of response to treatment include the sidedness of the cancer (right *vs* left), *RAS* and *BRAF* status, which affect prognosis in metastatic disease, and microsatellite instability status, which affects the outcome of adjuvant treatment.^23^ These questions cannot yet be answered by the results of SCOT, but there is a translational research substudy—the TransSCOT study, which is ongoing—that will look at these factors.

It is more difficult to give 6 months of treatment than 3 months of treatment. In the SCOT study, 2521 (83%) of 3024 patients assigned to 3 months received 3 months of treatment, whereas only 1773 (59%) of 3013 patients assigned to 6 months received 6 months of treatment. However, the median percentage of the full expected dose of fluoropyrimidine received varied between 94% and 98% for the 3 month group and 82% and 85% for the 6 month group, depending on regimen choice. The corresponding figures for oxaliplatin were between 96% and 98% for the 3 month group and 69% and 73% for the 6 month group. Given that the median fluoropyrimidine and oxaliplatin doses received were similar between the two regimens (figure 5), compliance to treatment is unlikely to explain the difference seen between the two regimens.

Our results showed that 6 months of adjuvant chemotherapy was significantly more toxic than 3 months of treatment. This difference was most marked for peripheral neuropathy, with 237 (58%) of 409 patients in the 6 month group reporting grade 2 or worse neuropathy compared with 103 (25%) of 420 patients in the 3 month group. Peripheral neuropathy, as reported via a patient questionnaire, was significantly worse in the 6 month group and persisted for at least 5 years (figure 7). Other adverse events that are symptomatically important to patients such as diarrhoea and hand-foot syndrome were also significantly more common with 6 months of treatment. We do note that our conclusions regarding long-term neuropathy must been seen in light of the substantial proportion of missing data. However, the effects are large, as are the significance levels, and there is no evidence that these comparisons are biased between the groups,

QoL, as measured by QLQ-C30 global health status and EQ-5D-3L, declined while patients were receiving chemotherapy. QoL was therefore predictably worse for longer in those patients assigned to receive 6 months of treatment compared with 3 months of treatment. QoL improved after treatment stopped and by 1 year, there were no clinically important differences between the two study groups.

It is now realised that clinician assessment of neuropathy with methods such as the CTCAE are less sensitive than patient-reported outcomes such as FACT/GOG-Ntx4 and EORTC QLQ chemotherapy-induced peripheral neuropathy (CIPN) 20.^24^ In this study, although QoL seemed to recover by 1 year, chronic sensory peripheral neuropathy persisted for up to 5 years. We found a significant difference in peripheral neuropathy, with more patients in the 6 month group reporting “Quite a bit“ or “Very much” numbness, tingling, or discomfort in their hands or feet in the FACT/GOG-Ntx4 questionnaire, which correlated with significantly worse QoL at 1 year, 3 years, and 5 years. These differences in QoL were at least minimally clinically important (apart from the result for EQ-5D visual analogue scale at 1 year). This observation is similar to one made in a study showing a correlation between those patients with the most neuropathy symptoms (worst 10%) measured by EORTC QLQ CIPN 20 and a reduction in QoL measured by QLQ-C30 between 2 years and 11 years after treatment for colorectal cancer with oxaliplatin.^25^ The absence of an overall difference in QoL seems to obscure a meaningful difference in the group of patients who have the worst long-term chronic neuropathy.

In a parallel initiative to SCOT, the IDEA collaboration was set up to consolidate the results from all trials worldwide that were attempting to answer this question of adjuvant chemotherapy duration, particularly for stage III colon cancer (ie, SCOT, TOSCA, IDEA France, CALGB/SWOG 80702, ACHIEVE, and HORG).^26^ Our results, like those from the IDEA collaboration^27^ suggest that the relative effect of shortening the duration of adjuvant chemotherapy might depend on the initial choice of chemotherapy regimen (CAPOX *vs* FOLFOX; p=0·069 for heterogeneity in SCOT). When we looked at the effect of duration for each regimen separately, CAPOX showed non-inferiority in terms of disease-free survival for 3 months versus 6 months of treatment, but this was not the case for patients receiving FOLFOX. The reasons for this difference are not clear. The choice of chemotherapy regimen was not randomised but instead chosen by the physician and patient and the reasons why specific regimens were chosen for individual patients are not known.

Also important to note for capecitabine, an oral drug that is self-administered at home, is that we only know the prescribed dose and do not have detailed compliance data, meaning that we might overestimate the fluoropyrimidine dose intensity for patients receiving CAPOX. However, in our experience, except in instances where clinicians curtail or modify doses according to intracycle toxicity, compliance with oral chemotherapy drugs is high.

Since the difference in the impact of treatment duration between the CAPOX and FOLFOX regimens does not seem to be easily explained by compliance with treatment or differences in the overall percentage of standard drug doses that were delivered, we perhaps need to consider other potential reasons for this finding. In the CAPOX regimen, the dose of oxaliplatin given with each cycle is higher than that given in FOLFOX, and therefore we presume that higher peak doses of oxaliplatin are achieved. Additionally, although the peak dose of fluoropyrimidine will be lower with CAPOX (capecitabine is given twice daily orally for two out of three weeks) than with FOLFOX (where the fluoropyrimidine is given as a bolus, then infused over 2 days every 2 weeks), the duration and continuity of exposure is greater. Could the micrometastatic disease be rendered more sensitive by one or both of these differences? If each of the cells in this micrometastatic setting are cycling through S phase only sporadically, does the continuity of exposure of the fluoropyrimidine in the form of capecitabine mean that there is a greater overall chance that these cells will be exposed to fluoropyrimidine at the critical part of the cell cycle, compared with fluorouracil given as bolus, then over 2 days every 2 weeks. This notion is supported by data from two previous adjuvant studies.^10^, ^28^ In terms of oxaliplatin, a drug that is nearly always given over 2–4 h, does the peak concentration have more of an effect than the frequency, giving an advantage to the CAPOX regimen and less attrition to efficacy when the overall duration of therapy is shortened? Additionally, a higher dose of oxaliplatin is given in the first 4 weeks of treatment with CAPOX (260 mg/m^2^) than with FOLFOX (170 mg/m^2^). It is very difficult to prove these speculative theories without developing appropriate adjuvant models of malignancy, but the results seen will certainly be a focus of strong debate over the coming months and years.

Stage III colorectal cancer is a heterogeneous disease and data from the IDEA collaboration and from multiple adjuvant trials have shown that patients with T1–3, N1 pathology have much better outcomes than those with either T4 or N2 features.^27^ This has led to the evolution of the concept of a high-risk stage III patient population, with either T4 or N2 disease, as opposed to the lower risk stage III population (T1–3, N1); we might need to consider whether patients with high-risk stage III disease need to be treated in a slightly different way to those with lower risk stage III disease.

Similarly, our results have shown that patients with T1–3, N1 disease have a much better 3 year disease-free survival than those with either T4 or N2 pathology. We found that, in patients with T1–3, N1 colorectal cancer, 3 months of treatment was non-inferior to 6 months (HR 0·908, 95% CI 0·75–1·098). However, for patients with T4 or N2 high-risk stage III disease, non-inferiority was not met (1·068, 0·934–1·222). The HR for disease-free survival is greater than 1 for the 3 months duration compared to 6 months, suggesting that there could be some small loss of efficacy, but the confidence intervals are wide, making this difference difficult to interpret with certainty. Notably, the observed absolute deficit in 3 year disease-free survival between the 3 months and 6 months of treatment for the high-risk group (T4 or N2 disease) is 1·8%, which has to be balanced against the increased toxicity seen with 6 months of treatment. This is particularly important because the worsened peripheral neuropathy persists for at least 5 years after treatment and results in worse QoL outcomes.

For patients with T4 or N2 pathology, the absolute increase in 3 year disease-free survival with 6 months versus 3 months of treatment was 2·7% (95% CI −4·1 to 9·6) for FOLFOX and 1·3% (95% CI −3·7 to 6·2) for CAPOX (appendix p 5). However, the forest plots did not show a difference in outcome according to duration of treatment between N1 and N2 disease. In view of the difference in toxicity seen with longer treatment, many patients will accept a small reduction in disease-free survival in exchange for reduced toxicity and this is especially true if they are able to receive CAPOX. There is less evidence to support a shorter duration of adjuvant treatment if it is decided that the patient needs to receive FOLFOX or has T4 disease.

Across the whole trial population, SCOT met the criteria for non-inferiority with a difference in 3 year disease-free survival of −0·4% (95% CI −2·6 to 1·8) between 3 months and 6 months of treatment. While the study was underpowered, the 95% CI for the HR lies below the non-inferiority boundary and the results are consistent with those of other individual studies by the IDEA group, taking into account how the duration effect depended on regimen and risk group. The consistency with these other studies indicates that the results of SCOT are unlikely to represent a false-positive in terms of showing non-inferiority. The concept that underpowered studies are more likely to produce false-positives (ignoring the factor of publication bias, which does not apply to a large scale enterprise such as SCOT) is disputed.^29^ As noted, the results differed with the (non-randomised) choice of chemotherapy regimens and we can recommend a 3 month duration of adjuvant chemotherapy for patients with T1–3, N1 disease (2677 [44%] of the 6088 patients in the SCOT study) if the patient is felt to be suitable for the CAPOX regimen. We have not been able to show with any statistical certainty that 3 months of treatment was non-inferior to 6 months for patients receiving FOLFOX, for whom there was an absolute increase in 3 year disease-free survival with 6 months versus 3 months of treatment of 2·9% (95% CI −6·7 to 0·8).

Despite the study's size, there are limitations to the reliability of conclusions that can be drawn for some subgroups. The choice of FOLFOX and CAPOX chemotherapy regimen was not randomised and there was clear heterogeneity between the two regimens, the reasons for which are unclear. For patients with low-risk stage 3 disease receiving CAPOX, 3 months of treatment is sufficient, whereas non-inferiority was not shown for FOLFOX. High-risk stage III disease includes either T4 or N2 disease (or both) and this study has not been able to show whether the effect of duration of adjuvant chemotherapy is the same for T4 and N2 disease. The final decision on treatment duration and regimen used for each individual will depend on a careful discussion between the clinician and patient, taking into account the risk of recurrence, the likely absolute difference in disease-free survival and risk of long-term toxicity, and the strength of evidence for that particular setting available both from SCOT and the wider IDEA analysis.

SCOT is, to our knowledge, the largest single randomised study on the adjuvant treatment of colorectal cancer to date. The study achieved its primary endpoint of showing that 3 months of oxaliplatin-containing adjuvant chemotherapy is non-inferior to 6 months of the same treatment in the overall trial population. 3 months of treatment might therefore be considered a new standard of care for adjuvant chemotherapy, especially if CAPOX is to be given.
