# Supplementary material for: 3 versus 6 months of adjuvant oxaliplatin-fluoropyrimidine combination therapy for colorectal cancer (SCOT): an international, randomised, phase 3, non-inferiority trial
Source: Lancet Oncol. 2018 Apr;19(4):562–78. doi: 10.1016/S1470-2045(18)30093-7 (PMC5883334; doi:10.1016/S1470-2045(18)30093-7)
Supplement: Supplementary appendix [file mmc1.pdf]

THE LANCET
Oncology

Supplementary appendix

This appendix formed part of the original submission and has been peer reviewed. We post it as supplied by the authors.

Supplement to: Iveson TJ, Kerr RS, Saunders MP, et al. 3 versus 6 months of adjuvant oxaliplatin-fluoropyrimidine combination therapy for colorectal cancer (SCOT): an international, randomised, phase 3, non-inferiority trial. *Lancet Oncol* 2018; **19:** 562–78.

Figure 1: Plot of 3 mnth/6 mnth hazard ratio by actual treatment duration* with associated non-inferiority p-values

Treatment duration	3 month arm Events/Patients	6 month arm Events/Patients	HR (95% CI) (3 mnth/6 mnth)
=3mnths v >3mnths	595 / 2513	532 / 2382	1.098 (0.977 - 1.234)
p= 0.31			
=3mnths v >4mnths	595 / 2513	489 / 2244	1.14 (1.011 - 1.285)
p= 0.55			
=3mnths v >5mnths	595 / 2513	435 / 2032	1.166 (1.03 - 1.32)
p= 0.69			
=3mnths v =6mnths	595 / 2513	380 / 1771	1.158 (1.018 - 1.317)
p= 0.64			
Overall	740 / 3035	742 / 3030	1.006 (0.909 - 1.114)
p= 0.012			

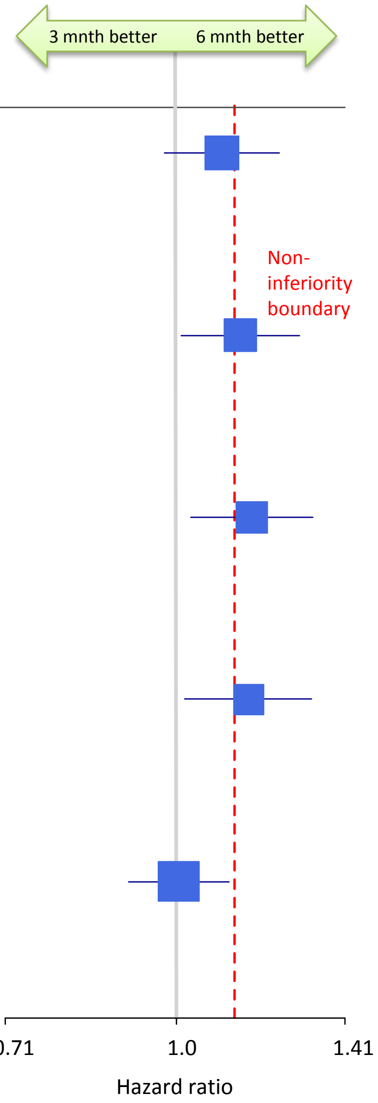

* Analysis according to duration restricted to eligible patients who started treatment

Figure 2: EORTC global health status by randomised study arm

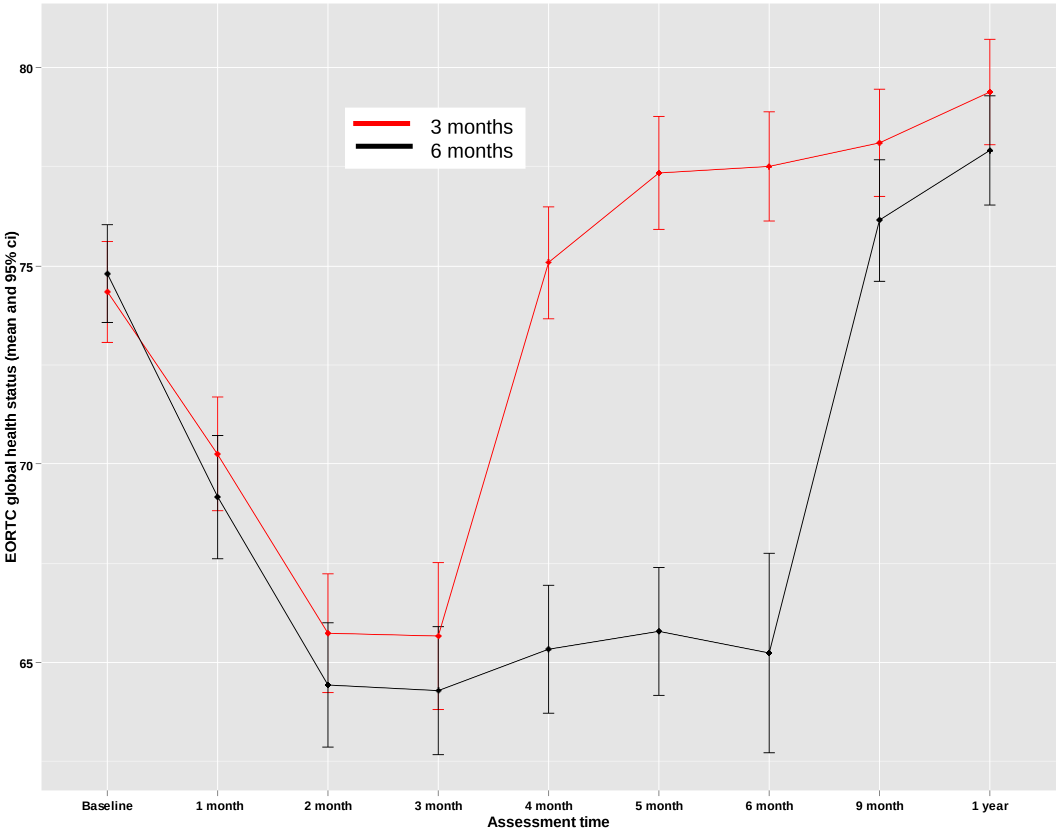

Number of patients completing questionnaire										
		Baseline	Month 1	Month 2	Month 3	Month 4	Month 5	Month 6	Month 9	Year 1
**three months of treatment**	N completed	796	719	660	456	543	522	509	642	593
	N expected	916	908	901	898	894	891	882	877	866
	%of those expected	87%	79%	73%	51%	61%	59%	58%	73%	68%
**six months of treatment**	N completed	794	677	664	597	554	504	204	554	582
	N expected	913	902	895	889	886	885	878	873	863
	%of those expected	87%	75%	74%	67%	63%	57%	23%*	63%582/916	67%

*Low return rate because patients assessed at start of last cycle rather than 6 months (which corresponds to end of cycle)

Figure 3: EQ-5D self-rated VAS by randomized study arm

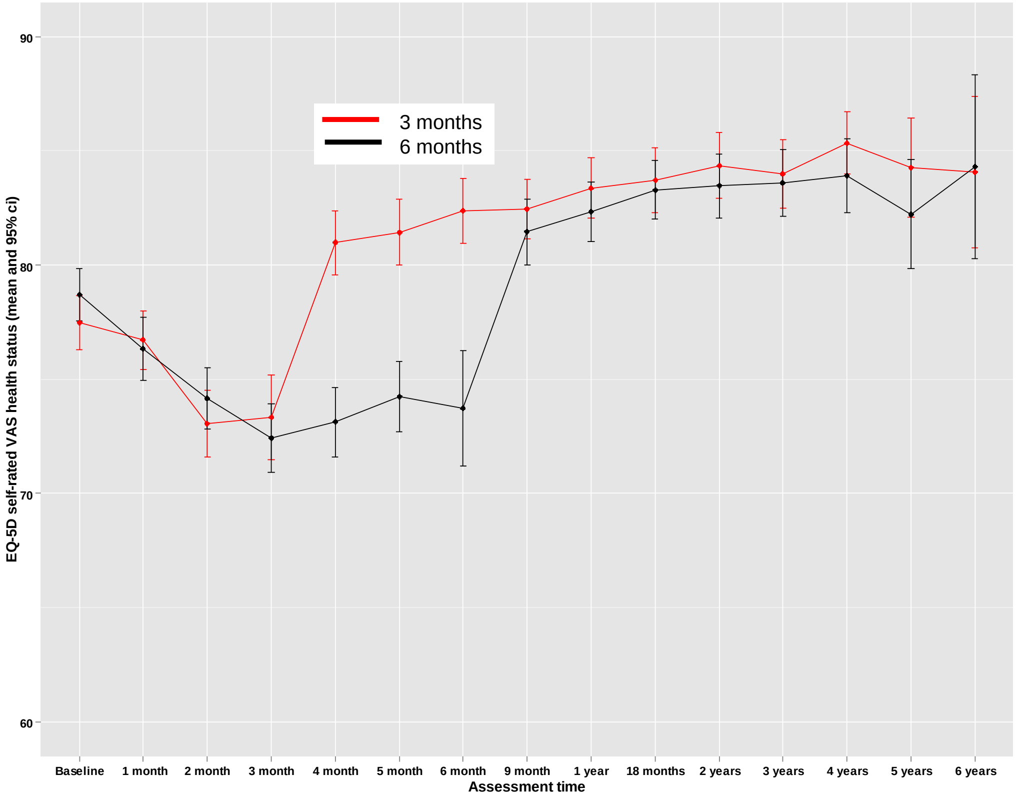

Number of patients completing questionnaire																
		Baseline	Month 1	Month 2	Month 3	Month 4	Month 5	Month 6	Month 9	Year 1	Mont 18	Year 2	Year 3	Year 4	Year 5	Year 6
three months of treatment	N completed	692	631	594	415	498	478	470	571	541	528	497	423	362	197	59
	N expected	916	907	901	898	894	891	882	877	865	842	807	752	588	317	84
	%of those expected	76%	70%	66%	46%	56%	54%	53%	65%	63%	63%	62%	56%	62%	62%	70%
six months of treatment	N completed	695	599	591	543	509	457	186	497	541	512	492	408	341	191	58
	N expected	912	902	895	889	886	884	878	873	862	848	815	761	617	319	86
	%of those expected	76%	66%	66%	61%	57%	52%	21%*	57%	63%	60%	60%	54%	55%	60%	67%

*Low return rate because patients assessed at start of last cycle rather than 6 months (which corresponds to end of cycle)
Note that although results are available beyond year 6 they have been omitted because of small numbers and resultant wide confidence intervals

Figure 4: Difference in quality of life scales between patients with "Quite a bit"/"Very much" numbness, tingling or discomfort in hands or feet on GOG NTX4 toxicity questionnaire and those who rated these symptoms as being present “Somewhat”/”A little bit” or “Not at all”

	EQ-5D self rated VAS	EQ-5D 3L Health index	EORTC global quality of life
1 Year	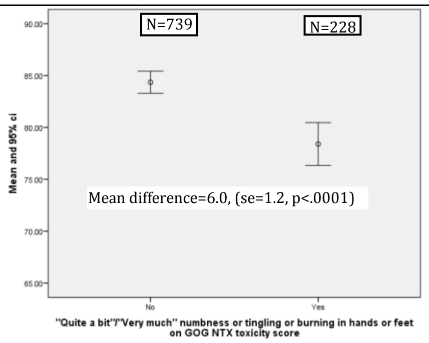	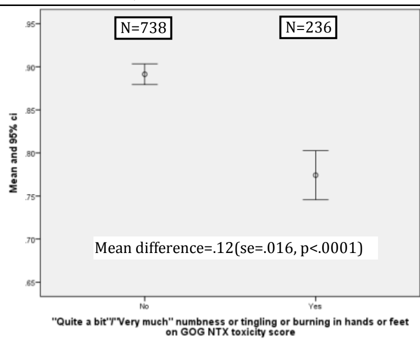	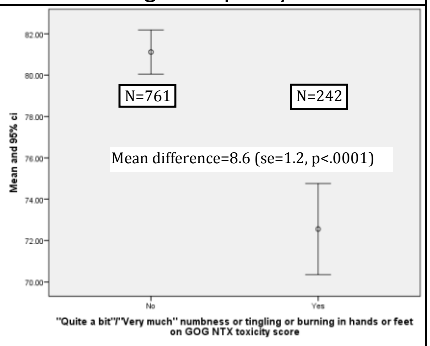
3 Years	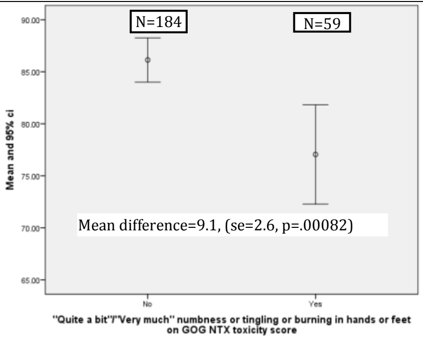	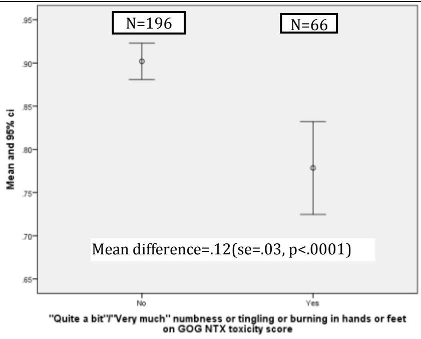	
5 Years	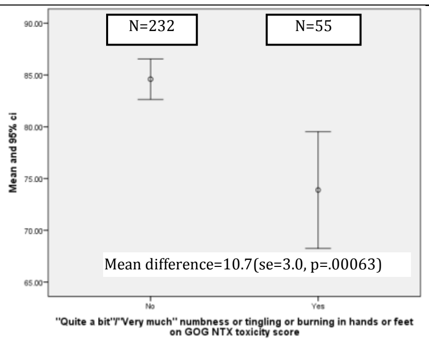	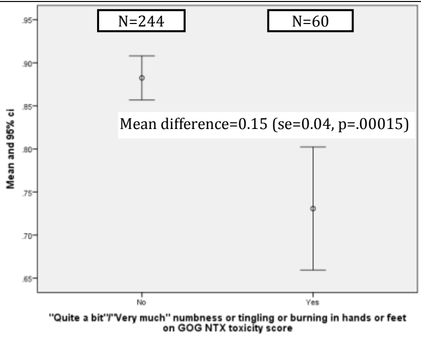	

Figure 5: Kaplan-Meier plots of disease-free by study arm, regimen and stage III risk group

Low risk stage III (T1-3/N1)

High risk stage III (T4 or N2)

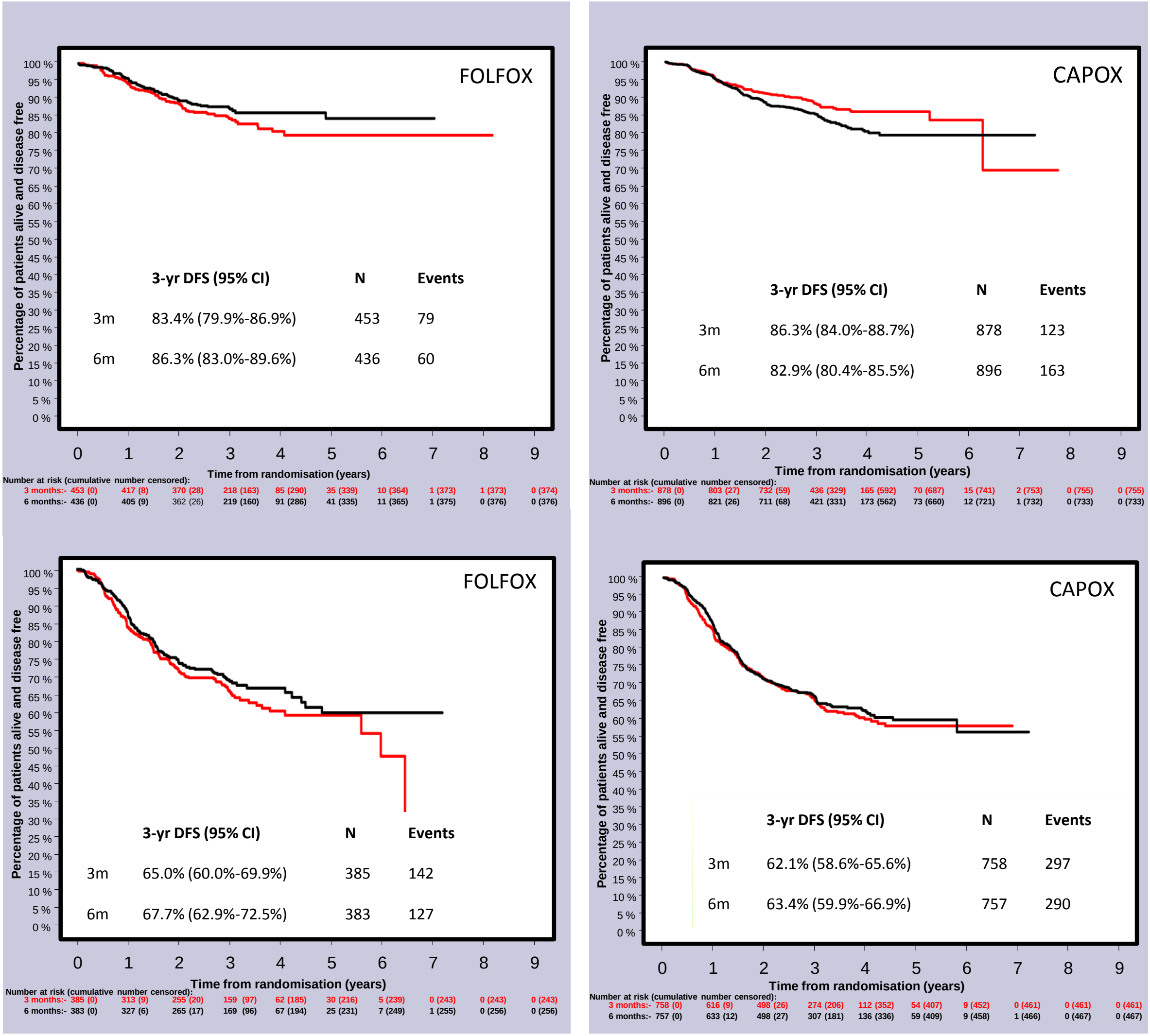

Figure 6: Percent grade 3/4/5 diarrhoea. hand-foot syndrome and neutropenia by study arm, and regimen

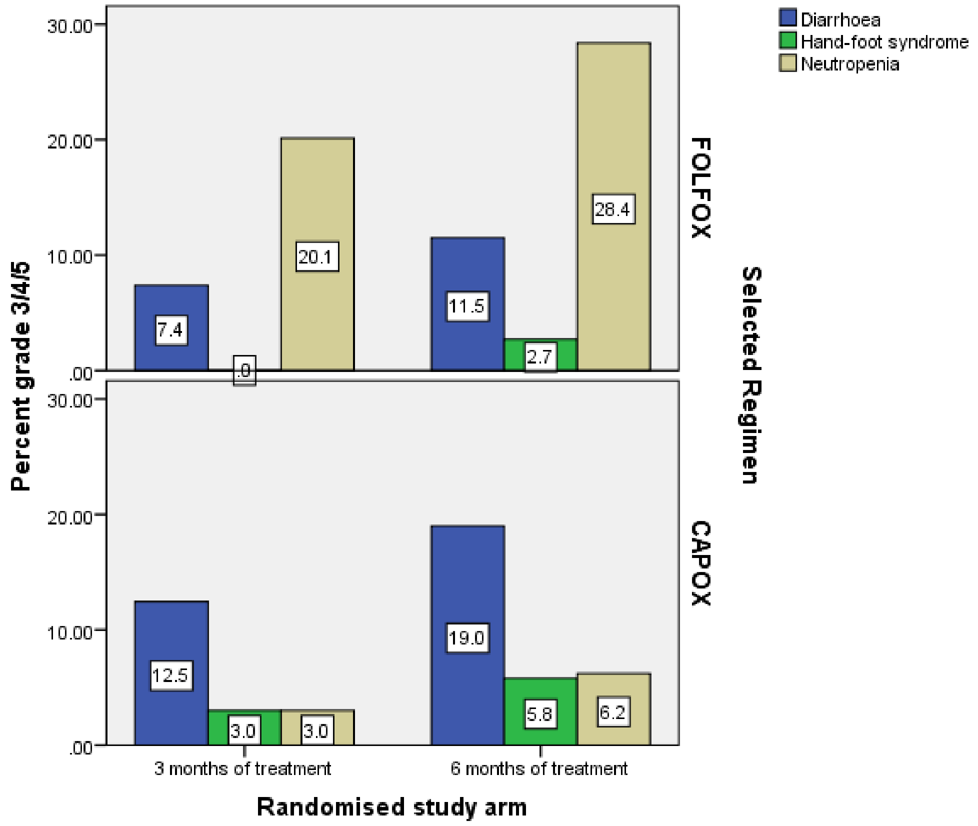

Table 1a: Patient characteristics for patients assessed by NCI CTC AE

		Randomised study arm			
		3 months of treatment		6 months of treatment	
		Column N %	Count	Column N %	Count
Gender	F	38.2%	166	38.0%	165
	M	61.8%	268	62.0%	269
	Total	100.0%	434	100.0%	434
Age at registration	Median	63		65	
	Interquartile-range	59-69		58-70	
	Range	27-83		20-58	
	N=	434		434	
Performance status at randomisation	0	63.6%	276	60.6%	263
	1	36.4%	158	39.4%	171
	Total	100.0%	434	100.0%	434
Disease site	Colon	81.6%	354	81.8%	355
	Rectum	18.4%	80	18.2%	79
	Total	100.0%	434	100.0%	434
T-stage	0	0.0%	0	0.2%	1
	1	2.5%	11	2.3%	10
	2	9.9%	43	9.0%	39
	3	56.7%	246	57.6%	250
	4	30.9%	134	30.9%	134
	Total	100.0%	434	100.0%	434
N-stage	0	13.4%	58	12.9%	56
	1	57.6%	250	58.8%	255
	2	29.0%	126	28.3%	123
	Total	100.0%	434	100.0%	434
Planned treatment	FOLFOX	35.7%	155	36.4%	158
	CAPOX	64.3%	279	63.6%	276
	Total	100.0%	434	100.0%	434
If CAPOX planned, starting dose of Capecitabine	750 mg/m2	27.8%	5	22.7%	5
	800 mg/m2	0.0%	0	0.0%	0
	1000 mg/m2	72.2%	13	77.3%	17
	Total	100.0%	18	100.0%	22
High risk stage II	No	86.9%	377	87.1%	378
	Yes	13.1%	57	12.9%	56
	Total	100.0%	434	100.0%	434

Table 1b Patient characteristics for patients assessed on EQ-5D

		Randomised study arm			
		3 months of treatment		6 months of treatment	
		Column N %	Count	Column N %	Count
Gender	F	39.4%	361	39.8%	363
	M	60.6%	555	60.2%	549
	Total	100.0%	916	100.0%	912
Age at registration		64		65	
		59-70		59-70	
		24-83		20-83	
		916		912	
Performance status at randomi-sation	0	65.0%	595	63.7%	581
	1	35.0%	321	36.3%	331
	Total	100.0%	916	100.0%	912
Disease site	Colon	82.4%	755	82.2%	750
	Rectum	17.6%	161	17.8%	162
	Total	100.0%	916	100.0%	912
T-stage	0	0.0%	0	0.1%	1
	1	2.4%	22	2.7%	25
	2	9.2%	84	8.8%	80
	3	57.5%	527	56.6%	516
	4	30.8%	282	31.8%	290
	X	0.1%	1	0.0%	0
	Total	100.0%	916	100.0%	912
N-stage	0	15.3%	140	15.5%	141
	1	56.7%	519	56.7%	517
	2	28.1%	257	27.9%	254
	Total	100.0%	916	100.0%	912
Planned treatment	FOLFOX	31.9%	292	32.6%	297
	CAPOX	68.1%	624	67.4%	615
	Total	100.0%	916	100.0%	912
If CAPOX planned, starting dose of Capecitabine	750 mg/m2	22.3%	84	22.4%	83
	800 mg/m2	4.8%	18	4.9%	18
	1000 mg/m2	72.9%	274	72.8%	270
	Total	100.0%	376	100.0%	371
High risk stage II	No	84.9%	778	84.9%	774
	Yes	15.1%	138	15.1%	138
	Total	100.0%	916	100.0%	912

Table 1c: Patient characteristics for patients assessed on EORTC QLQ-C30/CR29

		Randomised study arm			
		3 months of treatment		6 months of treatment	
		Column N %	Count	Column N %	Count
Gender	F	39.4%	361	39.8%	363
	M	60.6%	555	60.2%	550
	Total	100.0%	916	100.0%	913
Age at registration	Median	64		65	
	Interquartile range	59-70		59-70	
	Range	24-83		30-83	
	N=	916		913	
Performance status at randomi-sation	0	65.0%	595	63.7%	582
	1	35.0%	321	36.3%	331
	Total	100.0%	916	100.0%	913
Disease site	Colon	82.4%	755	82.3%	751
	Rectum	17.6%	161	17.7%	162
	Total	100.0%	916	100.0%	913
T-stage	0	0.0%	0	0.1%	1
	1	2.4%	22	2.7%	25
	2	9.2%	84	8.8%	80
	3	57.5%	527	56.6%	517
	4	30.8%	282	31.8%	290
	X	0.1%	1	0.0%	0
	Total	100.0%	916	100.0%	913
N-stage	0	15.3%	140	15.4%	141
	1	56.7%	519	56.7%	518
	2	28.1%	257	27.8%	254
	Total	100.0%	916	100.0%	913
Planned treatment	FOLFOX	31.9%	292	32.5%	297
	CAPOX	68.1%	624	67.5%	616
	Total	100.0%	916	100.0%	913
If CAPOX planned, starting dose of Capecitabine	750 mg/m2	22.3%	84	22.6%	84
	800 mg/m2	4.8%	18	4.8%	18
	1000 mg/m2	72.9%	274	72.6%	270
	Total	100.0%	376	100.0%	372
High risk stage II	No	84.9%	778	84.9%	775
	Yes	15.1%	138	15.1%	138
	Total	100.0%	916	100.0%	913

Table 1d Patient characteristics for patients assessed on GOG-NTX4

		Randomised study arm			
		3 months of treatment		6 months of treatment	
		Column N %	Count	Column N %	Count
Gender	F	38.9%	562	38.4%	548
	M	61.1%	883	61.6%	878
	Total	100.0%	1445	100.0%	1426
Age	Median	64		65	
	Interquartile-range	58-70		59-70	
	Range	24-83		20-85	
	N=	1445		1426	
Performance status at randomi-sation	0	68.7%	993	67.7%	965
	1	31.3%	452	32.3%	461
	Total	100.0%	1445	100.0%	1426
Disease site	Colon	80.9%	1169	81.0%	1155
	Rectum	19.1%	276	19.0%	271
	Total	100.0%	1445	100.0%	1426
T-stage	0	0.0%	0	0.1%	1
	1	2.4%	35	2.9%	42
	2	9.2%	133	8.8%	126
	3	57.5%	831	56.5%	806
	4	30.8%	445	31.6%	451
	X	0.1%	1	0.0%	0
	Total	100.0%	1445	100.0%	1426
N-stage	0	16.1%	232	16.1%	230
	1	57.4%	830	57.2%	816
	2	26.5%	383	26.6%	380
	Total	100.0%	1445	100.0%	1426
Planned treatment	FOLFOX	32.3%	467	32.1%	458
	Capox	67.7%	978	67.9%	968
	Total	100.0%	1445	100.0%	1426
If CAPOX planned, starting dose of Capecitabine	750 mg/m2	21.8%	159	20.6%	149
	800 mg/m2	3.8%	28	4.1%	30
	1000 mg/m2	74.4%	543	75.3%	545
	Total	100.0%	730	100.0%	724
High risk stage II	No	84.1%	1215	84.1%	1199
	Yes	15.9%	230	15.9%	227
	Total	100.0%	1445	100.0%	1426

Table 2: Causes of death

		Randomised study arm			
		3 months of treatment		6 months of treatment	
		Count	Column N %	Count	Column N %
Cause of death	N/A - Alive	2642	87.1%	2636	87.0%
	Colorectal cancer	277	9.1%	274	9.0%
	Protocol treatment related	16	.5%	16	.5%
	Other cancer	22	.7%	20	.7%
	Other treatment related	1	.0%	1	.0%
	Other causes	46	1.5%	48	1.6%
	Unknown	31	1.0%	35	1.2%
	Total	3035	100.0%	3030	100.0%

Table 3: Details of protocol treatment related causes of death

			Patient ID	Description of protocol treatment related cause of death
Randomised study arm	3 months of treatment	1	100	SEPTIC SHOCK AND MULTI-ORGAN FAILURE
		2	414	PULMONARY EMBOLISM
		3	550	CARDIAC ARREST
		4	1147	NEUTROPENIC SEPSIS, MULTI ORGAN FAILURE
		5	1585	PULMONARY FIBROSIS POSSIBLY RELATED TO OXALIPLATIN
		6	1995	POST MORTEM RESULT - ISCHEMIC HEART DISEASE
		7	2036	MYOCARDIAL INFARCTION RELATED TO CAPECITABINE.
		8	2767	PORTAL VEIN THROMBOSIS
		9	2886	MULTIPLE ORGAN FAILURE SECONDARY TO NEUTROPENIC SEPSIS
		10	3174	PULMONARY EMBOLISM
		11	3675	CARDIAC ARREST, CORONARY ARTERY SPASM RELATED TO CAPECITABINE
		12	3945	MULTI ORGAN FAILURE
		13	4077	PULMONARY EMBOLISM
		14	4285	FOUND DEAD AT HOME - NO POST MORTEM CARRIED OUT
		15	5355	CARDIAC ISCHEMIA INFARCTION
		16	5921	ISCHAEMIC BOWEL
		Total N	16	16
	6 months of treatment	1	385	SEPTIC SHOCK DUE TO NEUTROPENIA
		2	492	PULMONARY EMBOLISM, DEEP VEIN THROMBOSIS
		3	562	CAUSE OF DEATH IS CHEMOTHERAPY FOR RECTAL ADENOCARCINOMA
		4	1419	PULMONARY FIBROSIS SECONDARY TO OXALIPLATIN
		5	1532	DIARRHOEA + NEUTROPENIA RESULTING IN MULTI ORGAN FAILURE
		6	1771	MYOCARDIAL INSUFFICIENCY? CAPECITABINE RELATED
		7	1856	INTRACRANIAL BLEED
		8	2175	1A) ARRHYTHMIA, 1B) CHEMOTHERAPY INFUSION FOR COLON CANCER
		9	3752	MYOCARDIAL INFARCTION
		10	4200	MULTI-ORGAN FAILURE DUE TO AORTIC THROMBOSIS
		11	4258	MULTI-ORGAN FAILURE
		12	4721	ASPIRATION PNEUMONIA AND TYPE II DIABETES
		13	4846	PERFORATION
		14	5168	BILATERAL PULMONARY EMBOLISM
		15	5629	DIARRHOEA CAUSE BY CAPECITABINE INDUCED COLITIS
		16	5702	MYOCARDIAL INFARCTION
		Total N	16	16
	Total	N	32	32

Table 4: Serious adverse reactions by body system (only for events that effect >1% of patients in either arm)

	Randomised study arm				
	3 months of treatment		6 months of treatment		
Body System	Column N %	Count	Column N %	Count	P-value
BLOOD/BONE MARROW	1.5%	45	1.8%	54	.42
CARDIAC GENERAL	1.6%	48	1.4%	42	.53
CONSTITUTIONAL SYMPTOMS	3.3%	100	3.5%	104	.83
GASTROINTESTINAL	7.9%	238	8.6%	260	.33
INFECTION	2.4%	72	2.9%	86	.30
METABOLIC/LABORATORY	1.2%	35	1.2%	35	1.00
NEUROLOGY	1.2%	36	1.5%	46	.32
PAIN	2.2%	65	3.1%	94	.024
PULMONARY/UPPER RESPIRATORY	0.9%	27	1.5%	45	.043
VASCULAR	1.3%	38	2.1%	64	.012
Total	100.0%	3009	100.0%	3013	

Table 5: Maximum NCI CTC grade of toxicity recorded during treatment by randomised study arm (for toxicities with an overall incidence <10%, but at least one toxicity >3)

	Randomised study arm																							
	3 months of treatment (n=434)												6 months of treatment (n=434)											
	0		1- 2		3		4		5		Missing		0		1-2		3		4		5		Missing	
	Count	Row Valid N %	Count	Row Valid N %	Count	Row Valid N %	Count	Row Valid N %	Count	Row Valid N %	Count	Row Valid N %	Count	Row Valid N %	Count	Row Valid N %	Count	Row Valid N %	Count	Row Valid N %	Count	Row Valid N %	Count	Row Valid N %
Allergic reaction	419	99.8%	0	0.0%	1	0.2%	0	0.0%	0	0.0%	14	-	399	97.6%	8	2.0%	2	0.5%	0	0.0%	0	0.0%	25	-
Allergy - Other (Specify)	419	99.8%	1	0.2%	0	0.0%	0	0.0%	0	0.0%	14	-	406	99.3%	1	0.2%	2	0.5%	0	0.0%	0	0.0%	25	-
ALT	411	97.9%	9	2.1%	0	0.0%	0	0.0%	0	0.0%	14	-	392	95.8%	16	3.9%	1	0.2%	0	0.0%	0	0.0%	25	-
Ascites	420	100.0%	0	0.0%	0	0.0%	0	0.0%	0	0.0%	14	-	408	99.8%	0	0.0%	1	0.2%	0	0.0%	0	0.0%	25	-
Bilirubin	413	98.3%	7	1.7%	0	0.0%	0	0.0%	0	0.0%	14	-	400	97.8%	8	2.0%	1	0.2%	0	0.0%	0	0.0%	25	-
Blood - Other (Specify)	418	99.5%	2	0.5%	0	0.0%	0	0.0%	0	0.0%	14	-	403	98.5%	5	1.2%	1	0.2%	0	0.0%	0	0.0%	25	-
Blurred vision	417	99.3%	3	0.7%	0	0.0%	0	0.0%	0	0.0%	14	-	400	97.8%	9	2.2%	0	0.0%	0	0.0%	0	0.0%	25	-
Bronchospasm	419	99.8%	1	0.2%	0	0.0%	0	0.0%	0	0.0%	14	-	407	99.5%	1	0.2%	1	0.2%	0	0.0%	0	0.0%	25	-
Cardiac General - Other (Specify)	417	99.3%	1	0.2%	2	0.5%	0	0.0%	0	0.0%	14	-	407	99.5%	1	0.2%	1	0.2%	0	0.0%	0	0.0%	25	-
Cardiac ischemia/infarction	419	99.8%	0	0.0%	1	0.2%	0	0.0%	0	0.0%	14	-	406	99.3%	1	0.2%	2	0.5%	0	0.0%	0	0.0%	25	-
Cardiopulmonary arrest	420	100.0%	0	0.0%	0	0.0%	0	0.0%	0	0.0%	14	-	408	99.8%	0	0.0%	0	0.0%	0	0.0%	1	0.2%	25	-
Cholecystitis	420	100.0%	0	0.0%	0	0.0%	0	0.0%	0	0.0%	14	-	408	99.8%	0	0.0%	1	0.2%	0	0.0%	0	0.0%	25	-
CNS ischemia	419	99.8%	0	0.0%	0	0.0%	1	0.2%	0	0.0%	14	-	409	100.0%	0	0.0%	0	0.0%	0	0.0%	0	0.0%	25	-
Colitis	418	99.5%	1	0.2%	1	0.2%	0	0.0%	0	0.0%	14	-	407	99.5%	2	0.5%	0	0.0%	0	0.0%	0	0.0%	25	-
Cough	414	98.6%	6	1.4%	0	0.0%	0	0.0%	0	0.0%	14	-	394	96.3%	14	3.4%	1	0.2%	0	0.0%	0	0.0%	25	-
Creatinine	418	99.5%	2	0.5%	0	0.0%	0	0.0%	0	0.0%	14	-	404	98.8%	4	1.0%	1	0.2%	0	0.0%	0	0.0%	25	-
Dehydration	414	98.6%	1	0.2%	5	1.2%	0	0.0%	0	0.0%	14	-	399	97.6%	3	0.7%	7	1.7%	0	0.0%	0	0.0%	25	-
Dermatitis - Select	420	100.0%	0	0.0%	0	0.0%	0	0.0%	0	0.0%	14	-	407	99.5%	1	0.2%	1	0.2%	0	0.0%	0	0.0%	25	-
Distension	416	99.0%	4	1.0%	0	0.0%	0	0.0%	0	0.0%	14	-	407	99.5%	2	0.5%	0	0.0%	0	0.0%	0	0.0%	25	-
Dizziness	411	97.9%	9	2.1%	0	0.0%	0	0.0%	0	0.0%	14	-	389	95.1%	18	4.4%	2	0.5%	0	0.0%	0	0.0%	25	-
Dysphagia	418	99.5%	2	0.5%	0	0.0%	0	0.0%	0	0.0%	14	-	404	98.8%	5	1.2%	0	0.0%	0	0.0%	0	0.0%	25	-
Dyspnea	403	96.0%	15	3.6%	1	0.2%	1	0.2%	0	0.0%	14	-	382	93.4%	22	5.4%	4	1.0%	1	0.2%	0	0.0%	25	-
Edema larynx	420	100.0%	0	0.0%	0	0.0%	0	0.0%	0	0.0%	14	-	408	99.8%	1	0.2%	0	0.0%	0	0.0%	0	0.0%	25	-
Edema: trunk/genital	419	99.8%	0	0.0%	1	0.2%	0	0.0%	0	0.0%	14	-	409	100.0%	0	0.0%	0	0.0%	0	0.0%	0	0.0%	25	-
Enteritis	420	100.0%	0	0.0%	0	0.0%	0	0.0%	0	0.0%	14	-	407	99.5%	1	0.2%	1	0.2%	0	0.0%	0	0.0%	25	-
Febrile neutropenia	417	99.3%	0	0.0%	1	0.2%	1	0.2%	1	0.2%	14	-	409	100.0%	0	0.0%	0	0.0%	0	0.0%	0	0.0%	25	-
Fever	415	98.8%	4	1.0%	1	0.2%	0	0.0%	0	0.0%	14	-	389	95.1%	20	4.9%	0	0.0%	0	0.0%	0	0.0%	25	-
Flatulence	413	98.3%	7	1.7%	0	0.0%	0	0.0%	0	0.0%	14	-	403	98.5%	5	1.2%	1	0.2%	0	0.0%	0	0.0%	25	-
Fracture	420	100.0%	0	0.0%	0	0.0%	0	0.0%	0	0.0%	14	-	408	99.8%	0	0.0%	1	0.2%	0	0.0%	0	0.0%	25	-
Gait/walking	420	100.0%	0	0.0%	0	0.0%	0	0.0%	0	0.0%	14	-	408	99.8%	0	0.0%	1	0.2%	0	0.0%	0	0.0%	25	-
Gastritis	420	100.0%	0	0.0%	0	0.0%	0	0.0%	0	0.0%	14	-	407	99.5%	1	0.2%	1	0.2%	0	0.0%	0	0.0%	25	-
GI - Other (Specify)	410	97.6%	8	1.9%	2	0.5%	0	0.0%	0	0.0%	14	-	392	95.8%	14	3.4%	3	0.7%	0	0.0%	0	0.0%	25	-
Heartburn	401	95.5%	19	4.5%	0	0.0%	0	0.0%	0	0.0%	14	-	387	94.6%	21	5.1%	1	0.2%	0	0.0%	0	0.0%	25	-
Hemorrhage GI - Select	414	98.6%	5	1.2%	1	0.2%	0	0.0%	0	0.0%	14	-	405	99.0%	4	1.0%	0	0.0%	0	0.0%	0	0.0%	25	-
Hiccoughs	416	99.0%	4	1.0%	0	0.0%	0	0.0%	0	0.0%	14	-	402	98.3%	7	1.7%	0	0.0%	0	0.0%	0	0.0%	25	-
Hyperglycemia	419	99.8%	0	0.0%	1	0.2%	0	0.0%	0	0.0%	14	-	408	99.8%	0	0.0%	1	0.2%	0	0.0%	0	0.0%	25	-
Hyperuricemia	419	99.8%	0	0.0%	1	0.2%	0	0.0%	0	0.0%	14	-	408	99.8%	1	0.2%	0	0.0%	0	0.0%	0	0.0%	25	-
Hypoalbuminemia	410	97.6%	9	2.1%	1	0.2%	0	0.0%	0	0.0%	14	-	402	98.3%	7	1.7%	0	0.0%	0	0.0%	0	0.0%	25	-
Hypocalcemia	417	99.3%	2	0.5%	0	0.0%	1	0.2%	0	0.0%	14	-	406	99.3%	3	0.7%	0	0.0%	0	0.0%	0	0.0%	25	-
Hypoglycemia	419	99.8%	1	0.2%	0	0.0%	0	0.0%	0	0.0%	14	-	407	99.5%	0	0.0%	2	0.5%	0	0.0%	0	0.0%	25	-
Hypokalemia	411	97.9%	6	1.4%	2	0.5%	1	0.2%	0	0.0%	14	-	398	97.3%	9	2.2%	1	0.2%	1	0.2%	0	0.0%	25	-
Hyponatremia	417	99.3%	1	0.2%	2	0.5%	0	0.0%	0	0.0%	14	-	402	98.3%	5	1.2%	2	0.5%	0	0.0%	0	0.0%	25	-
Hypophosphatemia	417	99.3%	1	0.2%	2	0.5%	0	0.0%	0	0.0%	14	-	408	99.8%	1	0.2%	0	0.0%	0	0.0%	0	0.0%	25	-
Hypotension	419	99.8%	1	0.2%	0	0.0%	0	0.0%	0	0.0%	14	-	409	100.0%	0	0.0%	0	0.0%	0	0.0%	0	0.0%	25	-
Infection - Other (Specify)	397	94.5%	20	4.8%	3	0.7%	0	0.0%	0	0.0%	14	-	377	92.2%	26	6.4%	6	1.5%	0	0.0%	0	0.0%	25	-
Infection (documented clinically) - Select	418	99.5%	1	0.2%	1	0.2%	0	0.0%	0	0.0%	14	-	405	99.0%	2	0.5%	2	0.5%	0	0.0%	0	0.0%	25	-
Infection with normal ANC - Select	409	97.4%	7	1.7%	3	0.7%	1	0.2%	0	0.0%	14	-	397	97.1%	8	2.0%	4	1.0%	0	0.0%	0	0.0%	25	-
INR	418	99.5%	0	0.0%	2	0.5%	0	0.0%	0	0.0%	14	-	409	100.0%	0	0.0%	0	0.0%	0	0.0%	0	0.0%	25	-
Insomnia	401	95.5%	19	4.5%	0	0.0%	0	0.0%	0	0.0%	14	-	384	93.9%	25	6.1%	0	0.0%	0	0.0%	0	0.0%	25	-
Iron overload	420	100.0%	0	0.0%	0	0.0%	0	0.0%	0	0.0%	14	-	409	100.0%	0	0.0%	0	0.0%	0	0.0%	0	0.0%	25	-

	Randomised study arm																							
	3 months of treatment (n=434)												6 months of treatment (n=434)											
	0		1- 2		3		4		5		Missing		0		1-2		3		4		5		Missing	
	Count	Row Valid N %	Count	Row Valid N %	Count	Row Valid N %	Count	Row Valid N %	Count	Row Valid N %	Count	Row Valid N %	Count	Row Valid N %	Count	Row Valid N %	Count	Row Valid N %	Count	Row Valid N %	Count	Row Valid N %	Count	Row Valid N %
Joint-effusion	419	99.8%	0	0.0%	0	0.0%	1	0.2%	0	0.0%	14	-	409	100.0%	0	0.0%	0	0.0%	0	0.0%	0	0.0%	25	-
Joint-function	419	99.8%	1	0.2%	0	0.0%	0	0.0%	0	0.0%	14	-	406	99.3%	2	0.5%	1	0.2%	0	0.0%	0	0.0%	25	-
Laryngeal nerve	417	99.3%	2	0.5%	1	0.2%	0	0.0%	0	0.0%	14	-	404	98.8%	5	1.2%	0	0.0%	0	0.0%	0	0.0%	25	-
Leukocytes	415	98.8%	4	1.0%	1	0.2%	0	0.0%	0	0.0%	14	-	402	98.3%	7	1.7%	0	0.0%	0	0.0%	0	0.0%	25	-
Mental status	420	100.0%	0	0.0%	0	0.0%	0	0.0%	0	0.0%	14	-	408	99.8%	0	0.0%	1	0.2%	0	0.0%	0	0.0%	25	-
Mood alteration - Select	405	96.4%	14	3.3%	1	0.2%	0	0.0%	0	0.0%	14	-	393	96.1%	12	2.9%	4	1.0%	0	0.0%	0	0.0%	25	-
Muscle weakness - Select	420	100.0%	0	0.0%	0	0.0%	0	0.0%	0	0.0%	14	-	404	98.8%	4	1.0%	1	0.2%	0	0.0%	0	0.0%	25	-
Neurology - Other (Specify)	411	97.9%	8	1.9%	1	0.2%	0	0.0%	0	0.0%	14	-	396	96.8%	12	2.9%	1	0.2%	0	0.0%	0	0.0%	25	-
Obstruction GI	420	100.0%	0	0.0%	0	0.0%	0	0.0%	0	0.0%	14	-	407	99.5%	1	0.2%	1	0.2%	0	0.0%	0	0.0%	25	-
Ocular - Other (Specify)	417	99.3%	3	0.7%	0	0.0%	0	0.0%	0	0.0%	14	-	402	98.3%	6	1.5%	1	0.2%	0	0.0%	0	0.0%	25	-
Personality	419	99.8%	1	0.2%	0	0.0%	0	0.0%	0	0.0%	14	-	408	99.8%	0	0.0%	1	0.2%	0	0.0%	0	0.0%	25	-
Phlebitis	414	98.6%	6	1.4%	0	0.0%	0	0.0%	0	0.0%	14	-	396	96.8%	12	2.9%	1	0.2%	0	0.0%	0	0.0%	25	-
Pneumonitis	420	100.0%	0	0.0%	0	0.0%	0	0.0%	0	0.0%	14	-	407	99.5%	1	0.2%	1	0.2%	0	0.0%	0	0.0%	25	-
Psychosis	418	99.5%	1	0.2%	1	0.2%	0	0.0%	0	0.0%	14	-	409	100.0%	0	0.0%	0	0.0%	0	0.0%	0	0.0%	25	-
Pulmonary - Other (Specify)	413	98.3%	4	1.0%	2	0.5%	1	0.2%	0	0.0%	14	-	401	98.0%	7	1.7%	0	0.0%	1	0.2%	0	0.0%	25	-
Renal - Other (Specify)	418	99.5%	2	0.5%	0	0.0%	0	0.0%	0	0.0%	14	-	406	99.3%	2	0.5%	1	0.2%	0	0.0%	0	0.0%	25	-
Renal failure	416	99.0%	0	0.0%	4	1.0%	0	0.0%	0	0.0%	14	-	409	100.0%	0	0.0%	0	0.0%	0	0.0%	0	0.0%	25	-
Supraventricular arrhythmia - Select	418	99.5%	2	0.5%	0	0.0%	0	0.0%	0	0.0%	14	-	406	99.3%	2	0.5%	1	0.2%	0	0.0%	0	0.0%	25	-
Syncope (fainting)	419	99.8%	0	0.0%	1	0.2%	0	0.0%	0	0.0%	14	-	407	99.5%	0	0.0%	2	0.5%	0	0.0%	0	0.0%	25	-
Syndromes - Other (Specify)	419	99.8%	0	0.0%	1	0.2%	0	0.0%	0	0.0%	14	-	409	100.0%	0	0.0%	0	0.0%	0	0.0%	0	0.0%	25	-
Thrombosis/embolism (vascular access)	413	98.3%	1	0.2%	6	1.4%	0	0.0%	0	0.0%	14	-	407	99.5%	0	0.0%	1	0.2%	1	0.2%	0	0.0%	25	-
Thrombosis/thrombus/embolism	410	97.6%	2	0.5%	3	0.7%	4	1.0%	1	0.2%	14	-	396	96.8%	1	0.2%	3	0.7%	8	2.0%	1	0.2%	25	-
Tinnitus	417	99.3%	2	0.5%	1	0.2%	0	0.0%	0	0.0%	14	-	408	99.8%	1	0.2%	0	0.0%	0	0.0%	0	0.0%	25	-
Urinary frequency	420	100.0%	0	0.0%	0	0.0%	0	0.0%	0	0.0%	14	-	406	99.3%	3	0.7%	0	0.0%	0	0.0%	0	0.0%	25	-
Vascular - Other (Specify)	417	99.3%	2	0.5%	1	0.2%	0	0.0%	0	0.0%	14	-	403	98.5%	4	1.0%	1	0.2%	1	0.2%	0	0.0%	25	-
Vasovagal episode	419	99.8%	0	0.0%	1	0.2%	0	0.0%	0	0.0%	14	-	408	99.8%	1	0.2%	0	0.0%	0	0.0%	0	0.0%	25	-
Voice changes	420	100.0%	0	0.0%	0	0.0%	0	0.0%	0	0.0%	14	-	407	99.5%	1	0.2%	1	0.2%	0	0.0%	0	0.0%	25	-
Weight loss	414	98.6%	6	1.4%	0	0.0%	0	0.0%	0	0.0%	14	-	401	98.0%	7	1.7%	1	0.2%	0	0.0%	0	0.0%	25	-
Wound complication non-infectious	419	99.8%	0	0.0%	1	0.2%	0	0.0%	0	0.0%	14	-	408	99.8%	1	0.2%	0	0.0%	0	0.0%	0	0.0%	25	-

Table 6: Differences between study arms in selected[1] quality of life scales at month 6 and month 12

Scale	Standardised adjusted AUC p-value (p-value adjusted for multiple testing)	Difference at 6 months[2] (95% ci)	Difference at 12 months[2] (95% ci)
EORTC QLQ C30 Global Health Status			
Global Health Status	p<.0001 (p<.0001)	11.58 (9.62-13.54)	1.48 (-.19 -3.14)
EORTC QLQ C30 functional scales			
Cognitive	p<.0001 (p<.0001)	7.10 (5.09 – 9.10)	2.05 (.31 – 3.79)
Emotional	p<.0001 (p<.0001)	7.77 (5.71 – 9.83)	1.56 (-.19 – 3.32)
Physical	p<.0001 (p<.0001)	8.53 (6.43 – 10.63)	1.47 (.04 – 2.90)
Role	p<.0001 (p<.0001)	17.47 (14.94 -20.00)	3.79 (1.71 – 5.87)
Social	p<.0001 (p<.0001)	11.80 (8.59 – 15.01)	1.97 (0.29 – 3.64)
EORTC QLQ C30 symptom scales			
Fatigue	p<.0001 (p<.0001)	-15.31 (-18.48,-12.15)	-2.10 (-3.93,-.27)
Nausea and vomiting	p<.0001 (p<.0001)	-7.00 (-8.70, -5.30)	-.57 (-1.41,0.26)
Pain	p<.0001 (p<.0001)	-6.52 (-8.41, -4.63)	-.43 (-2.10, 1.24)
Dyspnoea	p=.00053 (p=.012)	-6.82 (-10.00, -3.64)	0.59 (-1.61, 2.79)
Insomnia	p<.0001 (p=.00014)	-8.78 (-11.15, -6.41)	-.32 (-3.10, 2.46)
Appetite loss	p<.0001 (p<.0001)	-14.86 (-17.08, -12.64)	-.08 (-1.35, 1.20)
Constipation	p=.0014 (p=.030)	-6.52 (-9.12, -3.93)	-.01 (-1.68, 1.67)
Diarrhoea	P<.0001 (p=.00025)	-7.72 (-9.78, -5.86)	-.34 (-2.09, 1.41)
Financial problems	p<.0001 (p=.00053)	-6.29 (-9.50, -3.08)	-.78 (-2.93, 1.37)
EORTC QLQ CR29			
Body image	p=.0019 (p=.037)	-5.42 (-7.24, -3.60)	-.66 (-2.43, 1.11)
Hair loss	p=.0017 (p=.035)	-7.78 (-10.89, -4.66)	-.73 (-2.19, .73)
Dry mouth	p<.0001 (p<.0001)	-17.69 (-20.97, -14.41)	-.08 (-1.35, 1.20)
Taste	p<.0001 (p<.0001)	-25.71 (-28.58, -22.83)	-.67 (-2.23, .90)
EQ-5D			
Visual analogue scale	p=.00081 (p=.00081)	9.80 (7.76 – 11.84)	1.45 (-.27 – 3.18)
Health index	p=.00056 (p=.00081)	0.102 (.069 - .135)	.015 (-.009, .039)

1. Those where the difference in standardised adjusted AUC was statistically significant at 5% after adjustment for multiple testing
2. 3 month arm-6 month arm

Table 7: Reasons for missing questionnaires as recorded on study CRF

a) EORTC Questionnaire

	ARM			
	3 months		6 months	
	Count	Column N %	Count	Column N %
Complete	6629	81.4%	7420	80.2%
Patient refused	175	2.1%	168	1.8%
Patient relapsed/evidence of new tumour	56	0.7%	53	0.6%
Patient unfit	33	0.4%	46	0.5%
Other	1233	15.1%	1542	16.7%
Not required	19	0.2%	28	0.3%

b) EQ5D Questionnaire

	ARM			
	3 months		6 months	
	Count	Column N %	Count	Column N %
Complete	8924	76.2%	9684	75.3%
Patient refused	256	2.2%	273	2.1%
Patient relapsed/evidence of new tumour	213	1.8%	181	1.4%
Patient unfit	73	0.6%	89	0.7%
Other	2204	18.8%	2573	20.0%
Not required	46	0.4%	55	0.4%

c) GOG NTX4 Questionnaire

	ARM			
	3 months		6 months	
	Count	Column N %	Count	Column N %
Complete	9658	64.4%	10858	65.6%
Patient refused	263	1.8%	281	1.7%
Patient relapsed/evidence of new tumour	190	1.3%	187	1.1%
Patient unfit	74	0.5%	101	0.6%
Other	2890	19.3%	3255	19.7%
Not required	1924	12.8%	1871	11.3%

It can be seen that the reasons are broadly similar between the study arms with the most common reason being “other”. Examination of a random sample of the free text on the CRF’s when the reason for “other” was entered indicates that in the vast majority of cases this was an error of some sort (form not returned, site error, patient forgot etc.). The high proportion of “not required” for GOG-NTX4 is due to administrative delay in implementing the amendment to extend the recording period for the questionnaire.

Table 8: Missing questionnaire (Yes/No) by baseline factors

		EORTC Missing				EQ-5D Missing				GOG-NTX4 Missing			
		No		Yes		No		Yes		No		Yes	
		Count	Row N %	Count	Row N %	Count	Row N %	Count	Row N %	Count	Row N %	Count	Row N %
Gender	F	5371	79.8%	1362	20.2%	7103	74.9%	2376	25.1%	7753	64.4%	4286	35.6%
	M	8678	81.3%	1991	18.7%	11505	76.2%	3587	23.8%	12763	65.4%	6750	34.6%
Age-group	<=50	1326	81.3%	304	18.7%	1715	75.1%	570	24.9%	1940	65.4%	1026	34.6%
	<=70	9529	80.9%	2250	19.1%	12666	76.2%	3958	23.8%	13961	65.0%	7503	35.0%
	>70	3194	80.0%	799	20.0%	4227	74.7%	1435	25.3%	4615	64.8%	2507	35.2%
Performance status	0	9259	80.6%	2222	19.4%	12190	75.4%	3980	24.6%	14112	65.4%	7451	34.6%
	1	4790	80.9%	1131	19.1%	6418	76.4%	1983	23.6%	6404	64.1%	3585	35.9%
T-stage	X/0/1	406	77.3%	119	22.7%	536	74.5%	183	25.5%	583	63.9%	330	36.1%
	2	1278	81.2%	296	18.8%	1710	76.8%	518	23.2%	1888	65.6%	991	34.4%
	3	8082	81.7%	1811	18.3%	10783	76.8%	3250	23.2%	11866	65.7%	6200	34.3%
	4	4283	79.2%	1127	20.8%	5579	73.5%	2012	26.5%	6179	63.7%	3515	36.3%
N-stage	0	2121	82.5%	451	17.5%	3072	77.2%	908	22.8%	3566	66.3%	1810	33.7%
	1	8004	81.1%	1863	18.9%	10514	76.2%	3287	23.8%	11724	65.5%	6172	34.5%
	2	3924	79.1%	1039	20.9%	5022	74.0%	1768	26.0%	5226	63.1%	3054	36.9%
Treatment	FOLFOX	5479	82.1%	1197	17.9%	6935	76.8%	2090	23.2%	7897	67.7%	3765	32.3%
	CAPOX	8570	79.9%	2156	20.1%	11673	75.1%	3873	24.9%	12619	63.4%	7271	36.6%

The table above shows the %-age missing for the PRO questionnaires across baseline patient characteristics. It can be seen that the percentage missing is very similar across the categories of the baseline characteristics with the difference being in general very small and never exceeding 5%.

Table 9: Sensitivity analysis of PRO results

	Imputed (as in manuscript)<br>*Mean (se)*	Observed data only<br>*Mean (se)*	Imputed (restricted to patients who completed baseline questionnaire)<br>*Mean (se)*
GOG NTX questionnaire			
Difference# at 6 months	-1.99 (.17)**	-2.31 (.35)**	-2.16 (.28)**
Difference at 12 months	-2.48 (.15)**	-2.54 (.23)**	-2.30 (.20)**
Difference at 5 years	-2.07 (.33)**	-1.90 (.42)**	-1.90 (.34)**
EORTC- QLQ-C30 global quality of life			
Difference at 6 months	11.58 (1.00)**	12.00 (1.41)**	12.72 (1.82)**
Difference at 12 months	1.48 (.85)~	1.57 (.99)~	1.22 (.86)~
EQ-5D Visual Analogue Scale			
Difference at 6 months	9.80 (1.04)**	7.05 (1.52)**	7.36 (0.90)**
Difference at 12 months	1.45 (.88)~	1.68 (.96)~	1.31 (1.00)~

Difference 3 months -6 months; **P<.001. ~P>.05

The tables above summarises the comparison of the three most important PRO end-points at key time-points. The first column is taken from the imputation analysis as presented in the paper, the second column is based on observed data and the last column is based on an imputation restricted to patients who completed a baseline questionnaire. This last analysis was done to ensure that the analysis set could not be influenced by a patient's subsequent experience on the study (for example patients who should have completed at baseline but were missed and subsequently completed a follow-up form because they developed neurotoxicity are omitted from this analysis).

At all time-points the mean differences are similar and completely consistent in terms of statistical significance.

SCOT SITE PRINCIPAL INVESTIGATOR RECRUITMENT		
**SITE NAME**	**PRINCIPAL INVESTIGATOR(S)**	**TOTAL RE-CRUITMENT**
BEATSON WEST OF SCOTLAND CANCER CENTRE	PROFESSOR JIM CASSIDY<br>DR ASHITA WATERSON	141
ROYAL UNITED HOSPITAL BATH	DR GARETH REES<br>DR LOUISE MEDLEY<br>DR EMMA DE WINTON	111
ADDENBROOKES HOSPITAL	DR CHARLES WILSON	97
ROYAL CORNWALL HOSPITAL	DR RICHARD ELLIS	94
ROYAL SURREY COUNTY HOSPITAL	DR GARY MIDDLETON<br>DR SHARADAH ESSAPEN	93
CASTLE HILL HOSPITAL	DR AMANDEEP DHADDA	91
MOUNT VERNON HOSPITAL	DR ROB HUGHES	89
BRISTOL ONCOLOGY CENTRE	DR STEPHEN FALK	85
QUEENS HOSPITAL	DR SHERIF RAOUF	83
SOUTHAMPTON GENERAL HOSPITAL	DR TIMOTHY IVESON	81
WESTERN GENERAL HOSPITAL	DR LESLEY DAWSON	80
CHRISTIE HOSPITAL NHS TRUST	DR MARK SAUNDERS	77
SALISBURY DISTRICT HOSPITAL	DR TIMOTHY IVESON<br>DR ALAAELDIN SHABLAK	77
VELINDRE NHS TRUST HOSPITAL	DR ALISON BREWSTER	76
ST BARTHOLOMEWS HOSPITAL	DR DAVID PROPPER	74
QUEEN ALEXANDRA HOSPITAL	DR ANN O'CALLAGHAN	73
SJÆLLANDS UNIVERSITES HOSPITAL, NÆSTVED	DR NEILS HENRIK HOLLÄNDER	70
PRINCESS ALEXANDRA HOSPITAL	DR JOHN BRIDGEWATER	69
ROYAL MARSDEN HOSPITAL (SUTTON)	PROFESSOR DAVID CUNNINGHAM	67
LEICESTER ROYAL INFIRMARY	PROFESSOR WILL STEWARD<br>PROFESSOR ANNE THOMAS	59
WORCESTER ROYAL HOSPITAL	DR DAVID FARRUGIA<br>DR MARK CHURN	59
SINGLETON HOSPITAL	PROFESSOR JOHN WAGSTAFF	57
BIRMINGHAM HEARTLANDS HOSPITAL	DR IAN GEH<br>DR SHOBHIT BAIJAL	55
MAIDSTONE & TUNBRIDGE WELLS NHS TRUST	DR MARK HILL	55
ROYAL SHREWSBURY HOSPITALS NHS TRUST	DR SAIF AWWAD<br>DR ABEL ZACHARIAH	54
NORTHAMPTON GENERAL HOSPITAL NHS TRUST	DR KINNARI PATEL<br>DR CRAIG MACMILLAN<br>DR ROSHAN AGARWAL	53
RUSSELLS HALL HOSPITAL	PROFESSOR DAVID FERRY<br>DR SIMON GRUMETT	51
LINCOLN COUNTY HOSPITAL	DR TOM SHEEHAN<br>DR ZUZANA STOKES	50
FIFE HEALTH BOARD	DR CATRIONA MCLEAN	48
KING EDWARD VII HOSPITAL	DR MARCIA HALL<br>DR MAHER HADAKI	48
ROYAL HAMPSHIRE COUNTY HOSPITAL	DR VIRGINIA HALL<br>DR SANJAY RAJ<br>DR DAVID NOLAN	48
SUNDERLAND ROYAL HOSPITAL	DR ASHRAF AZZABI	48
WEST SUFFOLK HOSPITAL	DR ANNE MARGARET MOODY	48
HAMMERSMITH HOSPITAL	DR CHARLES LOWDELL	47
CHURCHILL HOSPITAL	DR ANDREW WEAVER	46

SCOT SITE PRINCIPAL INVESTIGATOR RECRUITMENT		
**SITE NAME**	**PRINCIPAL INVESTIGATOR(S)**	**TOTAL RE-CRUITMENT**
AARHUS UNIVERSITY HOSPITAL	DR RENÉ KRØJGAARD OLESEN	45
YEOVIL DISTRICT HOSPITAL	DR STEPHEN FALK<br>DR JULIE WALTHER<br>DR ERICA BEAUMONT<br>DR MATTHEW SEPHTON	43
KETTERING GENERAL HOSPITAL	DR CRAIG MACMILLAN<br>DR GUY FAUST<br>DR ROSHAN AGARWAL	42
CROSSHOUSE HOSPITAL	DR JEFFERY WHITE	41
MUSGROVE PARK HOSPITAL	DR JULIE WALTHER<br>DR CLARE BARLOW	41
WORTHING HOSPITAL	DR ANDREW WEBB	41
HINCHINGBROOKE HOSPITAL	DR CHERYL PALMER	40
GREAT WESTERN HOSPITAL	DR CLAIRE HOBBS<br>DR SARAH LOWNDES	39
HOSPITAL UNIVERSITARI VALL D HEBRON	DR ELENA ELEZ MEELEZ	39
MONKLANDS DISTRICT GENERAL HOSPITAL	DR ASHITA WATERSTON<br>DR CLINTON ALI<br>DR ANNE MCKILLOP	39
WYCOMBE HOSPITAL	DR ANDREW WEAVER	39
AALBORG HOSPITAL	DR JORGEN HANSEN<br>DR METTE YILMAZ	38
IPSWICH HOSPITAL	DR RUBIN SOOMAL<br>DR LIZ SHERWIN	38
PILGRIM HOSPITAL	DR TOM SHEEHAN<br>DR ZUZANA STOKES	38
RAIGMORE HOSPITAL	DR DAVID WHILLIS<br>DR NEIL MCPHAIL	38
ST JAMES'S UNIVERSITY HOSPITAL	DR DANIEL SWINSON	38
STEPPING HILL HOSPITAL	DR JURJEES HASAN	38
WESTON PARK HOSPITAL NHS TRUST	DR SIMON PLEDGE	38
WITHYBUSH GENERAL HOSPITAL	DR VALLIPURAM VIGNESWARAN	38
BELFAST CITY HOSPITAL	DR RICHARD WILSON	37
GLAN CLWYD HOSPITAL	DR SIMON GOLLINS	37
GUY'S AND ST THOMAS' NHS TRUST	DR NICHOLAS MAISEY	37
POOLE HOSPITAL NHS TRUST	DR TAMAS HICKISH<br>DR AMELIE HARLE	37
ROYAL BOURNEMOUTH HOSPITAL	DR TAMAS HICKISH	37
WREXHAM MAELOR HOSPITAL	DR SIMON GOLLINS	37
HILLERØD HOSPITAL	DR SVEND ERIK NIELSEN	36
QUEEN ELIZABETH HOSPITAL - KING'S LYNN	DR ATHAR AHMAD<br>DR GAIL HORAN	36
ST GEORGE'S HOSPITAL	DR FIONA LOFTS	36
UNIVERSITY COLLEGE LONDON HOSPITAL	DR JOHN BRIDGEWATER	36
YORK HOSPITAL	DR KIM LAST	36
NOTTINGHAM UNIVERSITY HOSPITAL NHS TRUST	DR ERIC BESSELL<br>DR VANESSA POTTER<br>DR GEORGINA WALKER	35
ROYAL BERKSHIRE HOSPITAL	DR JAMES GILDERSLEVE	35
WEXHAM PARK HOSPITAL	DR MARCIA HALL<br>DR MAHER HADAKI	35

SCOT SITE PRINCIPAL INVESTIGATOR RECRUITMENT		
**SITE NAME**	**PRINCIPAL INVESTIGATOR(S)**	**TOTAL RE-CRUITMENT**
SOUTH TYNESIDE DISTRICT HOSPITAL	DR SHRAF AZZABI	34
BROOMFIELD HOSPITAL	PROFESSOR SAAD TAHIR<br>DR GOPALAKRISHNAN SRINIVASAN	33
EASTBOURNE DISTRICT GENERAL	DR FIONA MCKINNA	33
ICO L´HOSPITALET	DR RAMON SALAZAR<br>DR GEMMA SOLER	33
TORBAY HOSPITAL	DR NANGI LO	33
CONQUEST HOSPITAL	DR TIMOTHY SEVITT	32
LUTON AND DUNSTABLE HOSPITAL	DR SUZANNAH MAWDSLEY<br>DR FAYE LIM<br>PROFESSOR PETER HOSKIN	32
ABERDEEN ROYAL INFIRMARY	DR LESLIE SAMUEL	31
BASINGSTOKE + NORTH HAMPSHIRE HOSPITAL	DR CHARLOTTE REES	31
NORTH MIDDLESEX UNIVERSITY HOSPITAL	DR JOHN BRIDGEWATER	31
QUEEN'S HOSPITAL BURTON	DR PRABIR CHAKRABORTI<br>DR PUGAZHENTHI PATTU<br>DR MAJUSHA KENI	31
BLACKPOOL VICTORIA HOSPITAL	DR SHABBIR SUSNERWALA<br>DR SIN CHONG LAU	30
CHELTENHAM GENERAL HOSPITAL	DR DAVID FARRUGIA	30
REGIONSHOSPITALET HERNING	DR NINA KELDSEN	30
DIANA PRINCESS OF WALES HOSPITAL	DR RAJARSHI ROY	29
DORSET COUNTY HOSPITAL	DR RICHARD OSBORNE<br>DR MAXINE FLUBACHER	29
CUMBERLAND INFIRMARY	DR JONATHAN J NICOLL	28
GLOUCESTERSHIRE ROYAL HOSPITAL	DR DAVID FARRUGIA	28
HEREFORD COUNTY HOSPITAL	DR NICK REED	28
RIGSHOSPITALET (RH)	DR LONE NØRGAARD	28
WANSBECK HOSPITAL	DR WERNER DOBROWSKY<br>DR SANDEEP SINGHAL	28
CHARING CROSS HOSPITAL	DR CHARLES LOWDELL<br>DR SUSAN CLEATOR	27
PETERBOROUGH HOSPITAL	DR KAREN MCADAM<br>DR ABIGAIL HOLLINGDALE	27
QUEEN ELIZABETH HOSPITAL - GATESHEAD	DR WERNER DOBROWSKY<br>DR FIONA MCDONALD<br>DR MARK KATORY	27
QUEEN ELIZABETH HOSPITAL - BIRMINGHAM	DR NEIL STEVEN	27
SCARBOROUGH HOSPITAL	DR MOHAN HINGORANI<br>DR PUGAZHENTHI PATTU	27
SOUTHEND HOSPITAL	DR DAVID TSANG	27
JAMES COOK UNIVERSITY HOSPITAL	DR NICHOLAS WADD	27
ALTNAGELVIN HOSPITAL	DR RUSSELL HOUSTON<br>DR CLAIRE HARRISON<br>DR SONALI DASGUPTA	26
SCUNTHORPE GENERAL HOSPITAL	DR ABDEL HAMID<br>DR LORCAN O’TOOLE	26
GOOD HOPE HOSPITAL	DR JOHN GLAHOLM<br>DR SHOBHIT BAIJAL	25
ROYAL SUSSEX COUNTY HOSPITAL	DR ANDREW WEBB	25
HOSPITAL DONOSTIA	DR ADELAIDA-LACASTA MUNOA	24

SCOT SITE PRINCIPAL INVESTIGATOR RECRUITMENT		
SITE NAME	PRINCIPAL INVESTIGATOR(S)	TOTAL RE-CRUITMENT
NEW CROSS HOSPITAL	DR MARK CHURN<br>DR SIMON GRUMMET	24
UNIVERSITY HOSPITAL OF NORTH STAFFORDSHIRE	DR FAWZI ADAB	24
COUNTESS OF CHESTER HOSPITAL	DR ADRIAN MOSS<br>DR SHAKER ABDALLAH<br>DR DALE VIMALACHANDRAN	23
UNIVERSITY HOSPITAL OF LEWISHAM	PROFESSOR GEORGE MIKHAEL<br>DR JESSICA BRADY	23
ROYAL DERBY HOSPITAL	DR RAJENDRA KULKARNI	23
MEDWAY MARITIME HOSPITAL	DR JEFF SUMMERS<br>DR CHRISTOS MIKROPOULOS	22
ROYAL PRESTON HOSPITAL	DR SHABBIR SUSNERWALA<br>DR DEBORAH WILLIAMSON	22
UNIVERSITY HOSPITAL OF NORTH DURHAM	DR FAREEDA COXON	22
COMPLEJO HOSPIT. UNIVERSITARIO DE SANTIAGO	DR RAFAEL LOPEZ	21
FORTH VALLEY ROYAL HOSPITAL	DR GHAZIA SHAIKH<br>DR SARANYA KAKUMANU<br>DR STEPHEN HARROW<br>DR ADNAN SHAUKAT<br>DR AISHA TUFAIL<br>DR DAWN STOREY	21
KENT AND CANTERBURY HOSPITAL	PROFESSOR ROGER JAMES<br>DR JULIA HALL<br>DR RAKESH RAMAN	21
BASILDON HOSPITAL	DR DAVID TSANG	20
CLATTERBRIDGE CENTRE FOR ONCOLOGY	DR DAVID SMITH<br>DR JULIE O'HAGAN	20
FREEMAN HOSPITAL	DR FAREEDA COXON	20
STOKE MANDEVILLE HOSPITAL	DR ANDREW WEAVER	20
DARLINGTON MEMORIAL HOSPITAL	DR FAREEDA COXON	19
HARROGATE DISTRICT HOSPITAL	DR KIM LAST	19
HOSPITAL DEL MAR	DR JOAQUIM BELLMUNT	19
QUEEN ELIZABETH HOSPITAL - LONDON	DR NICHOLAS MAISEY<br>DR ASAD QURESHI	19
ROYAL MARSDEN HOSPITAL (FULHAM ROAD LON-DON)	PROFESSOR DAVID CUNNINGHAM	19
SJÆLLANDS UNIVERSITES HOSPITAL, ROSKILDE	DR JIM STENFATT LARSEN	19
ST HELENS AND KNOWSLEY NHS TRUST	DR ERNEST MARSHALL	19
KAROLINSKA UNIVERSITY HOSPITAL	DR GISELA NAUCLER<br>DR JAN-ERIK FRÖDIN	18
LISTER HOSPITAL	DR ROB HUGHES	18
ST MARY'S HOSPITAL (IOW)	DR CHRISTOPHER BAUGHAN<br>DR HARISH REDDY	18
GLANGWILI GENERAL HOSPITAL	DR MARGARET WILKINS<br>DR THI MAU-DON PHAN	18
WILLIAM HARVEY HOSPITAL	PROFESSOR ROGER JAMES<br>DR JULIA HALL	18
BRONGLAIS GENERAL HOSPITAL	DR ALAN AXFORD<br>DR SAJID DURRANI<br>DR ELIN JONES	17

SCOT SITE PRINCIPAL INVESTIGATOR RECRUITMENT		
**SITE NAME**	**PRINCIPAL INVESTIGATOR(S)**	**TOTAL RE-CRUITMENT**
DARENT VALLEY HOSPITAL	DR ANDREW GAYA<br>DR HAZIF AL GURAFI<br>DR RIYAZ NAZEERUL HAQ SHAH	17
HAIRMYRES HOSPITAL	DR GRAINNE DUNN	17
KIDDERMINSTER HOSPITAL	DR MARK CHURN	17
THE ROYAL OLDHAM HOSPITAL	DR SAIFEE MULLAMITHA	17
AYR HOSPITAL	DR JEFFERY WHITE	16
CRAIGAVON AREA HOSPITAL	DR RICHARD PARK	16
ESBJERG HOSPITAL	DR BRITA BJERREGAARD	16
KING'S MILL HOSPITAL	DR IVO HENNIG<br>DR ELEANOR JAMES<br>DR ELIOT CHADWICK<br>DR CHRISTINA LOPEZESCCOLA	16
WARRINGTON AND HALTON HOSPITALS NHS TRUST	DR ADRIAN MOSS<br>DR AMY FORD	16
HERLEV HOSPITAL	DR KIRSTEN VISTISEN	15
HOSPITAL ARNAU DE VILLANOVA LLEIDA	DR ANTONIA SALUD	15
MACCLESFIELD DISTRICT GENERAL HOSPITAL	DR CATHERINE MCBAIN<br>DR GANESH RADHAKRISHNA	15
BRADFORD ROYAL INFIRMARY	DR CHRIS BRADLEY<br>DR ANDREW CONN	14
NORTH WEST LONDON HOSPITALS NHS TRUST	DR NICOLA ANYAMENE	14
ROYAL FREE HOSPITAL	DR ASTRID MAYER	14
SØNDERBORG HOSPITAL	DR JURIJ BOGOVIC	14
UNIVERSITY HOSPITAL OF NORTH TEES	DR DAVID WILSON	14
BENDIGO HOSPITAL	DR ROBERT BLUM	13
BISHOP AUCKLAND GENERAL HOSPITAL	DR NICK WADD<br>DR FAREEDA COXON	13
CENTRAL HOSPITAL VASTERAS	DR HENRY LETOCHA<br>DR ANDRZEJ PIWOWAR	13
CITY HOSPITAL TRUST	DR DANIEL REA<br>DR PANKAJ PUNIA	13
MANOR HOSPITAL	DR ANDREW HARTLEY<br>DR VICTORIA KUNENE	13
ROYAL HOBART HOSPITAL	DR RAY LOWENTHAL<br>DR DAVID BOADLE	13
ROYAL ALBERT EDWARD INFIRMARY	DR GREGORY WILSON<br>DR ELENA TAKEUCHI<br>DR FRANCISCA ELENA MARTI	13
WESTON GENERAL HOSPITAL	DR MARJORIE TOMLINSON<br>DR REUBEN WEST	13
AUSTIN HOSPITAL	DR NIALL TEBBUTT	12
BANKSTOWN - LIDCOMBE HOSPITAL	DR RAY ASGHARI	12
NAMBOUR GENERAL HOSPITAL	DR MICHELLE CRONK	12
QUEEN ELIZABETH THE QUEEN MOTHER HOSPITAL	PROFESSOR ROGER JAMES<br>DR JULIA HALL	12
BEDFORD HOSPITAL NHS TRUST	PROFESSOR ROBERT THOMAS	11
HOSPITAL UNIVERSITARIO DE CANARIAS	DR MARTA LLANOS	11
HOSPITAL CLINICO UNIVERSITARIO DE VALENCIA	DR ANDRES CERVANTES	11
ROYAL MARSDEN HOSPITAL (KINGSTON)	DR DAVID CUNNINGHAM	11
UPPSALA UNIVERSITY HOSPITAL	DR BENGT GLIMELIUS	11

SCOT SITE PRINCIPAL INVESTIGATOR RECRUITMENT		
**SITE NAME**	**PRINCIPAL INVESTIGATOR(S)**	**TOTAL RE-CRUITMENT**
WEST CUMBERLAND HOSPITAL	DR JONATHAN J NICOLL	11
WEST MIDDLESEX UNIVERSITY HOSPITALS	DR PIPPA RIDDLE	11
HOSPITAL CLINIC I PROVINCIAL DE BARCELONA	DR CRISTINA NADAL<br>DR ESTELA PINEDA	10
NORTH DEVON DISTRICT HOSPITAL	DR MARK NAPIER	10
SANDWELL GENERAL HOSPITAL	DR DANIEL REA<br>DR PANKAJ PUNIA	10
SOUTHPORT AND FORMBY DISTRICT GENERAL HOS-PITAL	DR ARTHUR SUN MYINT<br>DR NASIM ALI	10
ST MARY'S HOSPITAL LONDON	DR SUSAN CLEATOR	10
THE TWEED HOSPITAL	PROFESSOR EHTESHAM ABDI	10
YSBYTY GWYNEDD HOSPITAL	DR NICK STUART<br>DR CATHERINE BALE	10
BALLARAT HOSPITAL	DR GEOFF CHONG	9
CANBERRA HOSPITAL	PROFESSOR DESMOND YIP	9
CENTRALLASARETTET VAXJO	DR EVA FERNEBRO<br>DR ULRIKA PALENIUS	9
COMPLEJO HOSPITALARIO UNIVERSITARIO DE ALBA-CETE	DR CARMEN ALONSO LOPEZ	9
HOSPITAL UNIVERSITARIO MARQUES DE VALDECILLA	DR FERNANDO RIVERA-HERRERO	9
ST JOHN OF GOD HOSPITAL BUNBURY	DR MARTIN BUCK	9
AIREDALE GENERAL HOSPITAL	DR ANDREW CONN	8
AINTREE UNIVERSITY HOSPITALS	DR DAVID SMITH<br>DR JULIE HAGAN	8
ALFRED HOSPITAL	DR ANDREW HAYDON	8
AUCKLAND HOSPITAL	DR GEORGE LAKING	8
CONCORD REPATRIATION GENERAL HOSPITAL	PROFESSOR PHILIP BEALE	8
FLINDERS MEDICAL CENTRE	DR CHRIS KARAPETIS	8
HOSPITAL UNIVERSITARIO MADRID NORTE SANCHINARRO	DR ANTONIO CUBILLO	8
NORTH TYNESIDE GENERAL HOSPITAL	DR PHILIP ATHERTON	8
ROYAL DEVON AND EXETER HOSPITAL	DR MELANIE OSBORNE<br>DR MARK NAPIER	8
COUNTY HOSPITAL RYHOV	DR HELGA HAGMAN	7
HOSPITAL LA FE	DR JORGE APARICIO	7
HOSPITAL VIRGEN DE LA ARRIXACA	DR MIGUEL MARIN VERA	7
INVERCLYDE ROYAL HOSPITAL	DR STEPHEN HARROW<br>DR DAWN STOREY	7
ROYAL LANCASTER INFIRMARY	DR DAVID FYFE<br>DR DAVID EATON	7
ROYAL BRISBANE AND WOMEN'S HOSPITAL	DR MATTHEW BURGE	7
WHITTINGTON HOSPITAL	DR DANIEL HOCHHAUSER<br>DR PAULINE LEONARD	7
UNIVERSITY HOSPITAL OF HARTLEPOOL	DR DAVID WILSON	7
UNIVERSITY HOSPITAL LINKÖPING	DR MARIA ALBERTSSON	7
CABRINI HOSPITAL	DR ANDREW HAYDON	6
CALDERDALE AND HUDDERSFIELD NHS FOUNDA-TION TRUST	DR JO DENT	6
DUMFRIES AND GALLOWAY ROYAL INFIRMARY	DR LESLEY DAWSON	6
HOSPITAL MIGUEL SERVET	DR ANTONIO ANTON	6
HOSPITAL DE TXAGORRITXU	DR SEVERINA DOMINGUEZ	6

SCOT SITE PRINCIPAL INVESTIGATOR RECRUITMENT		
**SITE NAME**	**PRINCIPAL INVESTIGATOR(S)**	**TOTAL RE-CRUITMENT**
LEIGHTON HOSPITAL	DR CHAN TON<br>DR MICHAEL BRAUN	6
MILTON KEYNES GENERAL HOSPITAL	Dr JILL STEWART<br>DR GERARD ANDRADE<br>DR SOMNATH MUKHERJEE	6
PRINCE OF WALES HOSPITAL	PROFESSOR DAVID GOLDSTEIN	6
QUEEN ELIZABETH HOSPITAL (AUSTRALIA)	PROFESSOR TIM PRICE	6
ROYAL NORTH SHORE HOSPITAL	DR ALEX GUMINSKI<br>ASSOCIATE PROFESSOR NICK PAVLA-KIS	6
ST VINCENT'S HOSPITAL	DR EVA SEGELOV	6
ESSEX COUNTY HOSPITAL	DR BRUCE SIZER	5
FALU HOSPITAL	DR AKE BERGLUND	5
PINDERFIELDS GENERAL HOSPITAL	DR GIREESH KUMARAN<br>DR KONSTANTINOS KAMPOSIORAS	5
CAMPBELLTOWN HOSPITAL	DR LORRAINE CHANTRILL	4
CHRISTCHURCH HOSPITAL	DR MARK JEFFERY	4
FURNESS GENERAL HOSPITAL	DR ALISON BIRTLE<br>DR DAVID EATON	4
ILLAWARRA CANCER CENTRE (WOLLONGONG HOS-PITAL)	DR MORTEZA AGHMESHEH	4
KALMAR COUNTY HOSPITAL	DR CHARLOTTE BRATTHALL	4
NEVIL HALL HOSPITAL	DR NAYYER IQBAL<br>DR MOHAMMED HARB<br>DR KEITH YINN	4
NEPEAN CANCER CARE CENTRE	DR JENNY SHANNON	4
NORRLANDS UNIVERSITY HOSPITAL	DR BIRGITTA LINDH	4
PRINCESS ALEXANDRA HOSPITAL (AUSTRALIA)	DR WARREN JOUBERT	4
ROYAL DARWIN HOSPITAL	DR NARAYAN KARANTH	4
ST JOHN OF GOD HOSPITAL SUBIACO	DR SIOBHAN NG<br>DR TOM VAN HAGEN	4
TAMWORTH BASE HOSPITAL	DR MATHEW GEORGE	4
ANTRIM AREA HOSPITAL	DR COLIN PURCELL	3
ST RICHARD'S HOSPITAL	DR SUHAIL BALUCH<br>DR ANN O’CALLAGHAN<br>DR YASSER HABA	3
WELLINGTON HOSPITAL	DR ANNE O'DONNELL	3
BALLARAT ONCOLOGY AND HAEMATOLOGY SER-VICES	DR GEORGE KANNOURAKIS	2
COFFS HARBOUR HEALTH CAMPUS	DR KAREN BRISCOE	2
GOSFORD HOSPITAL	DR SUSAN TILEY	2
HALLANDS HOSPITAL	DR LOTTA LUNDGREN	2
ROYAL PERTH HOSPITAL	DR DAVID RANSOM	2
UNIVERSITY HOSPITAL OF SKÅNE	DR MARGARETA HEBY	2
ARMIDALE HOSPITAL	ASSOCIATE PROFESSOR NICK PAVLA-KIS	1
DUNEDIN HOSPITAL	DR CHRIS JACKSON	1
HOSPITAL DE CRUCES	DR GUILLERMO LOPEZ VIVANCO	1
ICO DEL HOSPITAL JOSEP TRUETA	DR BERNARDO QUERALT	1
ROYAL LIVERPOOL UNIVERSITY HOSPITAL	DR DAVID SMITH	1
SUNDSVALL HÄRNÖSAND COUNTY HOSPITAL	DR PETRA FLYGARE	1
